# Genotype and Agronomic Management Interactions Shape the Accumulation of Immunogenic and Toxic Gluten Peptides in Durum Wheat

**DOI:** 10.1021/acs.jafc.5c16192

**Published:** 2026-04-28

**Authors:** Giovanni Caccialupi, Leonardo Cicala, Justyna Milc, Alessandro Ulrici, Fatma Boukid, Arnaldo Dossena, Sara Graziano, Barbara Prandi, Giovanna Visioli, Nelson Marmiroli, Mariolina Gullì, Pasquale De Vita, Nicola Pecchioni, Enrico Francia

**Affiliations:** 1 Department of Life Sciences, 308313 University of Modena and Reggio Emilia, Via Amendola 2, Pad. Besta, Reggio Emilia 42122, Italy; 2 Department of Chemistry, Life Sciences and Environmental Sustainability, Centre SITEIA. PARMA, University of Parma, Parma 43124, Italy; 3 Research Centre for Cereal and Industrial Crops, CREA-CI, Foggia 71122, Italy

**Keywords:** durum wheat, gluten protein, immunogenic peptides, agronomic management

## Abstract

Currently, there is growing interest in wheat-based products with a reduced digestive impact. We investigated whether agronomic management could modulate the accumulation of immunogenic peptides in durum wheat by using two multifactorial field trials. In the first trial, immunogenic peptide levels were influenced by genotype and by its interaction with nitrogen supply and sowing density, indicating that management effects are highly genotype dependent. In the second trial, the sowing date and fertilization regime affected immunogenic peptide accumulation, with the sowing date being the main driver. Spring sowing reduced the grain yield and increased grain protein and immunogenic peptide levels compared with fall sowing. Overall, these results support the adoption of genotype-specific management strategies to reduce immunogenic peptide accumulation while maintaining a yield in durum wheat.

## Graphical abstract

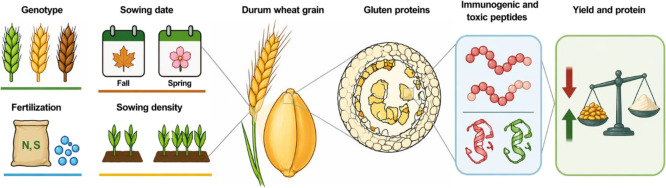

## Introduction

Durum wheat (*Triticum turgidum* L. subsp. *durum* (Desf.) Husn) is a key cereal crop worldwide and especially in Mediterranean agriculture. It is cultivated for the production of semolina used for making different kind of pasta, breads, couscous, and other traditional foods.^1^ The unique technological and end-use properties of durum wheat products are largely determined by gluten, which is composed of glutenins and gliadins. Glutenin proteins consist of different subunits of high molecular weight (HMW-GS, 80–140 kDa) and low molecular weight (LMW-GS, 31–51 kDa).^2^ Gliadins are monomeric alcohol-soluble proteins that can be classified into two categories: S-rich prolamins, i.e., α/β/γ-gliadins (molecular weight 36–44 kDa), and S-poor prolamins, i.e., ω-gliadins (molecular weight 44–78 kDa).^3^ When flours are hydrated, the gluten network gives dough its characteristic viscoelastic properties, with glutenins providing elasticity and gliadins conferring extensibility.^4^ The delicate balance and total content of these proteins are crucial for achieving high-quality food products and are influenced by a combination of genetics, environmental factors, and agronomic practices.^5,6^

For a growing number of individuals, gluten-related disorders pose a significant health challenge. The immunogenic potential of wheat represents a problem for many people who have a genetic predisposition to celiac disease or who suffer from nonceliac gluten sensitivity.^7^ The primary triggers are specific peptides from the α-gliadin protein family that resist full digestion and can provoke an immune response.^8^

In the context of celiac disease (CD), particular attention has been focused to α-gliadin peptides that resist gastrointestinal digestion and can act as toxic (TPT) or immunogenic (IPT) epitopes in genetically predisposed individuals.^9–11^ Toxic peptides are those capable of inducing mucosal damage when added in culture to duodenal mucosal biopsy or administered *in vivo* to the proximal and distal intestines; immunogenic peptides are those capable of stimulating specific T-cell lines and clones derived from the jejunal mucosa or peripheral blood of celiac patients. Studies have demonstrated the role of α-gliadin peptides acting as TPT and IPT epitopes in CD inducing a rapid damage to the intestinal mucosa^12^ and causing inflammatory reaction commonly in all patients,^13^ respectively. The genetic and physiological mechanisms behind the CD are partly understood; a life-long gluten-free diet is the only effective cure for celiac individuals, thus far.^14^

The concentration of these epitopes is not a fixed trait; it is a product of a complex interplay between genotype and environment.^15–17^ While durum wheat is generally considered to have a lower allergenic potential than hexaploid bread wheat, significant variation in the concentration of these harmful peptides still exists in the species, providing a clear opportunity for targeted research.^18,19^ The genotype of a wheat cultivar determines its inherent potential to produce storage-protein composition, which largely determine dough rheology through the balance between gliadins and glutenins.^19^ However, the final storage-protein profile is highly plastic and responsive to agronomic management.^20^ Among nutritional factors, nitrogen fertilization is a primary driver of grain protein concentration and can modify the partitioning among gluten fractions, often increasing gliadin accumulation and potentially the immunogenic load.^21^ Sulfur availability also contributes to shaping gluten composition because S limitation tends to depress S-rich storage proteins, with consequences for gluten quality and end-use performance.^22^

Environmental conditions can further amplify or mitigate the predisposition of a genotype toward specific protein profiles.^23,24^ In Mediterranean environments characterized by irregular rainfall, terminal drought, and rising temperatures, yield and quality traits are strongly shaped by Genotype × Environment (G × E) interactions, resulting in context-dependent gluten profiles.^25^ Importantly, changes in total protein content or in the gliadin/glutenin balance do not necessarily translate proportionally into the abundance of specific immunogenic epitopes, because epitope load depends on cultivar-specific protein sequences and their contribution to the digestible peptide pool.^26^

Despite the recognized influence of genotype, nutrition, and climate on grain protein composition, a critical gap remains in understanding how specific combinations of these factors translate into the final epitope profile. This study addresses this gap by investigating how genotype, sowing date, fertilization, and sowing density collectively impact durum wheat productivity and the accumulation of TPT and IPT epitopes. Through two independent field experiments, our goal is to investigate cultivar-specific responses and management strategies that can enhance the technological value and productivity while simultaneously lowering the concentration of potentially harmful peptides. The findings will provide a scientific framework for further studies, for developing both new breeding programs and more effective on-farm practices, aiming to a more gluten-aware food system.

## Materials and Methods

### Experimental Site and Meteorological Data

Field experiments were carried out during the 2016–2017 growing season, on a clay-loam soil (Typic Chromoxerert) at the experimental farm of CREA-CI Research Centre for Cereal and Industrial Crops, Foggia, Italy (41°27′44.9″ N 15°30′03.9″ E). Daily temperatures (°C) and rainfall accumulation (mm) were recorded at the meteorological station of the CREA-CI research Centre (CIG Z312891276), located within 300 m of the experimental fields. In Figure S1, seasonal weather patterns are summarized in monthly data compared with the long-term average ranging from 1953 to 2012. Monthly rainfall accumulation (mm), minimum and maximum temperatures (°C), and the total radiation of the 2016/2017 cropping season are available in Figures S2 and S3.

### Field Trials and Plant Materials

Two independent, multifactorial field experiments were conducted to investigate the effects of genotype, agronomic practices, and their interactions on the accumulation of gluten epitopes in durum wheat.

The first experiment was the “GDN” trial, designed to evaluate the interaction between Genotype, Sowing Density, and Nitrogen fertilization. This was set as a three-factor factorial experiment within a randomized complete block design (RCBD) with three replicates (Table S1). Eight durum wheat cultivars (*Triticum turgidum* L. subsp. *durum* (Desf.) Husn) were evaluated: Aureo, Cannizzo, Creso, Iride, Saragolla, Senatore Cappelli, Simeto, and Svevo (Additional Information in Table S2). Two sowing densities were used: 200 and 400 plants/m^2^. Nitrogen fertilizations were applied at three total seasonal rates: N0 = 0 kg of N/ha, N50 = 50 kg of N/ha, and N100 = 100 kg of N/ha. In N50 and N100, 50 kg N/ha were applied presowing using an NP (18–46) fertilizer. In the N100 treatment, the additional 50 kg N/ha was applied as topdressing at tillering (Zadoks GS 22 ^27^) as ammonium nitrate. The experiments were conducted by using a randomized complete block design with three replicates for each treatment. Each entry was sown in plots consisting of 8 rows 2 m long, spaced 0.17 m apart, with 50 cm between the plots.^19^

The second experiment was the “GSO” trial, designed for a focused investigation on the effects on two contrasted Genotypes of the Sowing date and Organic-fertilization type. For this trial, two specific cultivars, Cannizzo and Saragolla (*Triticum turgidum* L. subsp. *durum* (Desf.) Husn), were selected. This choice was based on previous findings,^19^ which demonstrated that these two genotypes exhibited differentiated behaviors regarding grain yield and quality parameters. This experiment was also a three-factor factorial (Genotype × Sowing Date × Fertilization) set within an RCBD with three replicates (Table S3). The varieties were grown in 10 m^2^ plots, each consisting of eight rows, 7.5 m long and spaced 0.17 m apart, and sown in two distinct periods: fall sowing (12/12/2016) and spring sowing (22/02/2017), with a sowing density of 350 seeds/m^2^. Three different fertilizer treatments were applied before sowing: Control (N0), Mineral (N–P 18–46), and Organic-mineral (12–25 Nutrigrantop S, supplied by SCAM S.p.a. company, Modena, Italy; Table S4). Mineral and organic-mineral fertilization were applied in three split doses at distinct phenological stages to reach a total of 100 kg N/ha: at sowing, as topdressing at the beginning of seedling growth (Zadoks GS 10 ^27^), and as topdressing at tillering (Zadoks GS 22 ^27^).

### Field Management and Data Collection

Standardized field management was applied according to best agronomic practices. Following the protocol of Taranto and coauthors,^19^ weeds were uniformly controlled in both field trials using the herbicides Tralcossidim (1.7 L/ha), Clopiralid + 2-methyl-4-chlorophenoxyacetic acid (MCPA) + Fluoroxypyr (2.0–2.5 L/ha). During the crop season, the number of days from sowing to heading (DtH, Growth Stage GS55, 50% of ears emerged; according to Zadoks et al.^27^) was registered. At the end of the growing season, plants were harvested after physiological maturity on June 19, 2017, to measure the grain yield at 13% moisture content (GY, t/ha). Kernels from the three-replication plots were used to determine the grain protein content (GPC, %) by NIR (Infratec 1241 Analyzer, Foss, Hillerod, Denmark).

### Sample Milling

For both field experiments, grains were sorted, eliminating damaged seeds, and cleaned by removing straw, chaff, and other threshing residues. A sample of 30 g of dried seeds from each cultivar/site/year combination was milled into a fine powder of whole meal semolina with a Knifetec 1095 (Foss, Hillero̷d, Denmark), applying three cycles of 10 s each. The coarse whole flour obtained was stored at −20 °C until subsequent analyses.^17^ Sodium dodecyl sulfate (SDS) sedimentation assay was assessed for all samples using a 2% sodium dodecyl sulfate solution in accordance with AACC method 56–70,^28^ and results were recorded in milliliters (mL). Carotenoid content of the wheat was assayed using the water saturated *n*-butanol extracts with spectroscopic measurements at 435.8 nm by the AACC method 14–50.^28^ Carotenoid content was included as an additional end-use quality trait because carotenoid pigments are the main determinants of the yellow color of durum wheat endosperm and semolina, a key quality attribute for semolina and pasta products.^29^

### In Vitro Digestion and LC-MS/MS Quantification

Whole wheat flours were subjected to simulated gastrointestinal digestion protocol.^30^ In brief, 1 g of flour was incubated for 2 min at 37 °C with gentle continuous mixing in 1 mL of simulated saliva containing porcine amylase (Sigma-Aldrich, St. Louis, Michigan, USA) at an activity of 75 U/mL of digesta. Next, 2 mL of simulated gastric juice with porcine pepsin (2000 U/mL of digesta; Sigma-Aldrich) was added, the pH was adjusted to 3, and the mixture was incubated for 2 h at 37 °C under constant gentle mixing. Subsequently, 4 mL of duodenal juice containing porcine pancreatin (100 U trypsin activity/mL of digesta; Sigma-Aldrich) and porcine bile (10 mmol/L in the total volume; Sigma-Aldrich) was introduced, the pH was raised to 7, and incubation continued for an additional 2 h at 37 °C with gentle mixing. To terminate enzymatic activity, samples were heated at 95 °C for 10 min. After centrifugation at 3220*g* for 45 min at 4 °C, 295 μL of the supernatant was combined with 5 μL of an internal standard solution (TQQPQQPF­(d5)­PQQPQQPF­(d5)­PQ; 1.6 mM).^31,32^ The prepared samples were then analyzed using reverse-phase ultraperformance liquid chromatography coupled with electrospray ionization mass spectrometry (RP-UPLC/ESI-MS) for the quantification of peptides related to celiac disease. Characteristic ions for each peptide were extracted, resulting in extracted ion chromatograms (XICs), in which the identified peptides and internal standard were integrated using MassLynx software. The quantification value was obtained as the ratio of the peptide area to the internal standard area multiplied by the molar amount of the internal standard. All digestions were performed in duplicate.^18^

### Sequential Extraction and Quantification of Gluten Proteins

Gluten proteins were fractionated by a sequential extraction procedure already described by Graziano et al.^16^ to obtain gliadins and glutenins (HMW-GS and LWM-GS) fractions. In total, 30 mg of flour for each durum wheat sample was processed in three technical replicates for each sample. After 1.5 mL of propan-2-ol 55% (v/v) was added and the mixture was mixed for 20 min at 65 °C, supernatants containing the majority of the gliadin fraction were recovered by centrifugation and vacuum-dried. This step was repeated twice to remove possible gliadin residues. The pellets, containing the glutenin subunit (GS) fraction, were resuspended in propan-2-ol 55% (v/v), 0.08 M Tris–HCl pH 8.3, and 1% dithiothreitol (DTT) (w/v). After incubation at 60 °C for 30 min with continuous mixing, the supernatants, containing HMW-GS and LMW-GS fractions, were recovered by centrifugation for 5 min at 14,000 rpm. A proper volume of acetone was added to each sample to reach a final concentration of 40% (v/v), which was incubated for 10 min at room temperature. After centrifugation, the supernatants, containing the LMW-GS fraction, were precipitated again with acetone (up to a final concentration of 80% (v/v)), whereas the pellets, containing the HMW-GS fraction, were air-dried. Finally, all the extracted gliadins, HMW- and LMW-GS were dissolved in 50% (v/v) acetonitrile (ACN) with 0.1% (v/v) trifluoroacetic acid (TFA). Relative quantification was done in triplicate by the Bradford assay, using the iMark microplate reader (Bio-Rad, Boston, Massachusetts, USA).

### Statistical Analysis

Based on the experimental designs reported in the Supporting Information (Tables S1 and S3), treatment effects were analyzed in GenStat (17th edition; VSN International, Hemel Hempstead, UK) using a three-way analysis of variance (ANOVA), assuming normality, homoscedasticity, and independence of residuals. When significant effects were detected, means were compared using Duncan’s multiple range test (*p* < 0.05) to identify statistically homogeneous groups. For ANOVA results, bar plot graphs were generated using GraphPad Prism software version 8.0.2 (GraphPad Software, San Diego, California, USA). The heatmap graph was developed by using R (version 4.4.2) using the packages: *tidyverse* ^33^ *ggplot2*,^33^ *viridis*,^34^ and *scales*.^35^ Multivariate relationships among grain yield and quality variables were investigated using R (version 4.4.2). Pairwise associations were quantified using Pearson’s correlation coefficients and visualized as a correlation heatmap. Principal component analysis (PCA) was then performed on the same standardized data set using mean-centering and unit-variance scaling (autoscaling) to prevent dominance of variables with larger variances. Based on the analysis of the variance explained by each component through scree plots, three PCs were selected for both trials. Score and loading plots were generated for PC1–PC2 and PC1–PC3. Graphics and associated tables were generated in R using packages including *tidyverse*,^33^ *ggplot2*,^33^ and *ggrepel.* ^36^

## Results

### GDN Trial

#### Three-Way Interaction: Genotype × Fertilization × Sowing Density

The results of the ANOVA (Supporting Information, Tables S5 and S6) showed a statistically significant three-way interaction between Genotype, nitrogen fertilization, and Sowing Density for immunogenic gluten peptieds (IPT, ppm), toxic gluten peptides (TPT, ppm), and grain yield (t/ha). This complex interaction highlights that both the effect of fertilization and density are strongly genotype-dependent.

To visualize these combinations, the performances of the 48 treatment combinations for IPT, TPT, and GY are presented in Figure 1. The analysis highlights the combined effect of genotype, nitrogen fertilization level, and sowing density on the accumulation of immunogenic gluten peptides (IPT), toxic gluten peptides (TPT), and grain yield (GY).

**Figure 1:**
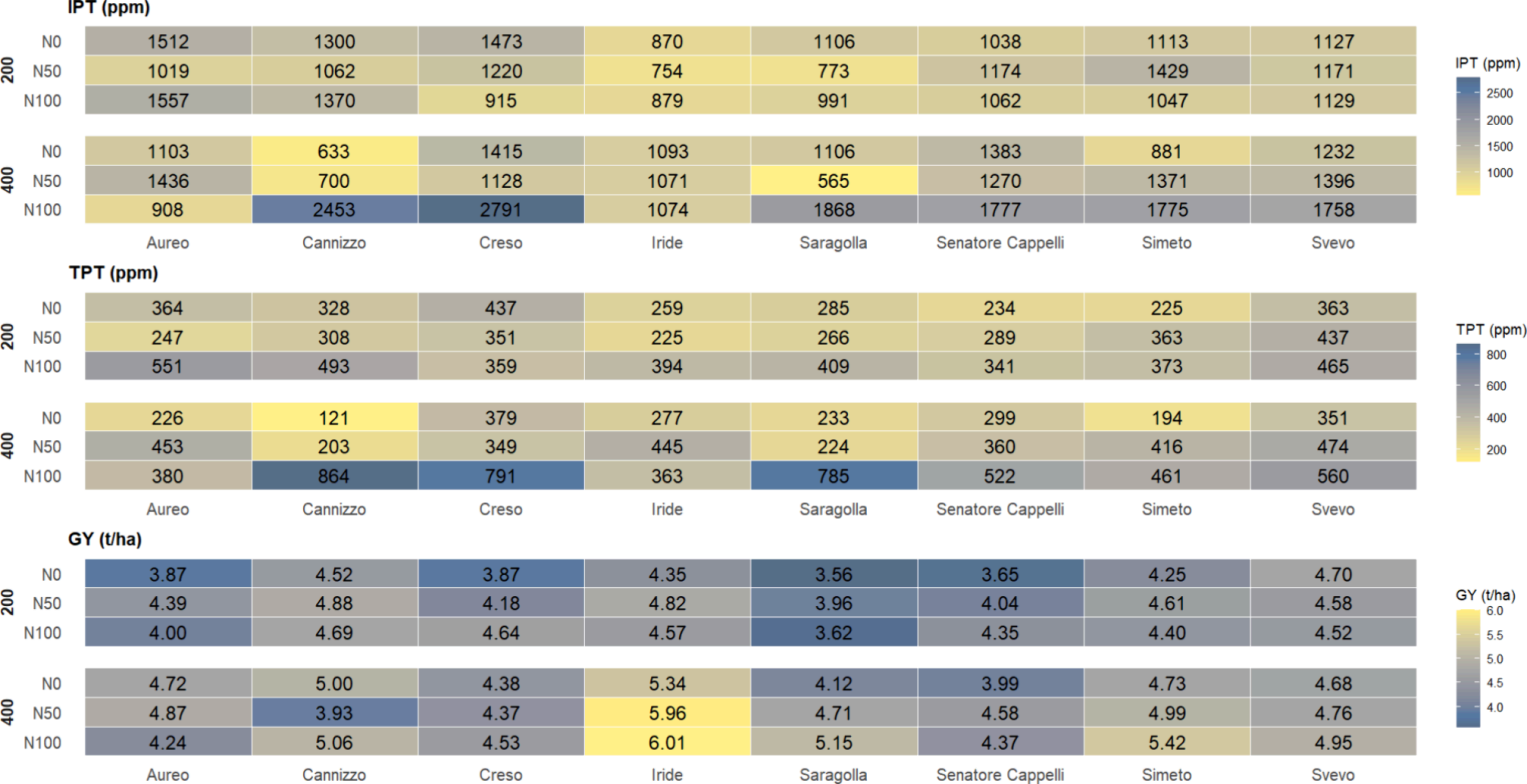
Heatmap summarizing the interaction between Genotype, nitrogen fertilization, and Sowing Density for IPT, TPT, and GY. For each genotype (column), mean values are reported for the six managements combinations obtained crossing the three levels of nitrogen fertilization (N0, N50, and N100) and the two levels of sowing density (200 and 400 plants/m^2^). Cell color intensity reflects the magnitude of the trait within each panel, with lower IPT and TPT values shown in lighter shades and higher GY values shown in lighter shades, to facilitate the visual identification of management combinations that maximize grain yield while minimizing gluten protein fractions. The ANOVA results and multicomparison by Duncan post hoc test are provided in Tables S5 and S6.

Specific combinations were identified that successfully balanced a high yield with a low peptide content. For instance, Saragolla under N50 fertilization at 400 plants/m^2^ achieved one of the lowest IPT concentrations (565 ppm) and a low TPT value (224.1 ppm). In the pedoclimatic conditions of the study, Cannizzo performed at its best with no nitrogen (N0) at 400 plants/m^2^, resulting in the lowest TPT (121.7 ppm) and high yield (5.0 t/ha). In contrast, Iride achieved its best balance (low IPT/TPT, high yield) at a lower density (200 plants/m^2^) with moderate nitrogen content (N50). Several other combinations showed comparably low IPT levels but differed in TPT and yield outcomes. For instance, Iride at 200 plants/m^2^ with high nitrogen (N100) showed one of the highest TPT levels (394.4 ppm) despite having moderate IPT.

Other noteworthy combinations included Cannizzo N50 200, Iride N0 400, and Aureo N0 400, all of which exhibited relatively high IPT values (1062, 1093, and 1103 ppm, respectively). TPT values were also elevated for Cannizzo N50 200 (308.2 ppm) and Iride N0 400 (277.8 ppm), while Aureo N0 400 had an intermediate TPT (226.1 ppm). In terms of yield, Cannizzo N50 200 and Aureo N0 400 showed similar performances (4.9 and 4.7 t/ha), whereas Iride N0 400 stood out with the highest yield across all combinations (5.3 t/ha).

These findings highlighted the complex interactions among genotype, nitrogen fertilization, and plant density in influencing grain yield and protein-related traits. Specific combinations with lower levels of fertilization (N0 and N50) and higher sowing density (400 plants/m^2^) emerged as particularly promising options for balancing high productivity with reduced levels of potentially immunogenic proteins. However, these results demonstrate that a single ″best practice″ for density or N fertilization does not exist; optimal management must be tailored to the specific genotype, in a specific environment. Therefore, preliminary management trials are suggested in a specific environment for a variety targeted by the supply chain, in order to obtain low-immunogenic peptides levels.

#### Multivariate Structure of Yield and Quality Traits in the GDN Trial: Pearson Correlations and PCA

The Pearson correlation matrix highlighted association patterns among the response variables (Figure S4). The immunogenicity indices were strongly intercorrelated (IPT vs TPT, *r* = 0.86; TITP vs IPT, *r* = 0.99; TITP vs TPT, *r* = 0.92), supporting their interpretation as a shared underlying dimension. Importantly, these indices were also positively associated with protein and gluten composition traits: IPT and TPT correlated with grain protein content (*r* = 0.35–0.45) and with total gluten protein content (*r* = 0.39–0.42). Immunogenicity indices showed positive correlations with gliadins (IPT vs gli, *r* = 0.34; TPT vs gli, *r* = 0.37; Total Immunogenic and Toxic Pepiteds TITP vs gli, *r* = 0.36), linking higher immunogenic peptide indices to the gluten fraction most enriched in immunogenic epitopes. Gluten-related traits were themselves strongly structured; notably, the total gluten protein content was strongly correlated with gliadins (gli, *r* = 0.94). In contrast, grain yield and phenology showed weak-to-moderate relationships with most quality traits (e.g., gy vs dth, *r* = −0.35). Carotenoids showed negligible correlations with the immunogenicity indices (|*r*| ≤ 0.18), indicating limited relevance to the IPT/TPT-related trait structure in this data set. Overall, the observed collinearity among several variables supported the use of principal component analysis (PCA) to summarize multivariate covariation patterns.

In the PCA model, three principal components were selected, with PC1 explaining 30.7%, PC2 18.5%, and PC3 15.2% of the total data variance (cumulative 64.4%). The PC1–PC2 score plot (49.2% of explained data variance) is reported in Figure 2B, where samples are colored according to nitrogen fertilization level, showing clearly that PC1 is heavily influenced by increasing nitrogen input. The corresponding PC1–PC2 loading plot (Figure 2A) shows that PC1 is primarily driven by the immunogenicity indices (TITP, IPT, and TPT) together with gluten traits, suggesting that higher N levels are associated with higher immunogenicity indices and a higher gluten protein content. A less marked but still significant effect of was also observed for sowing density and genotype, as reported in the relevant score plots (Figures S6–S10) and PC1–PC3 loading plot (Figure S5).

**Figure 2:**
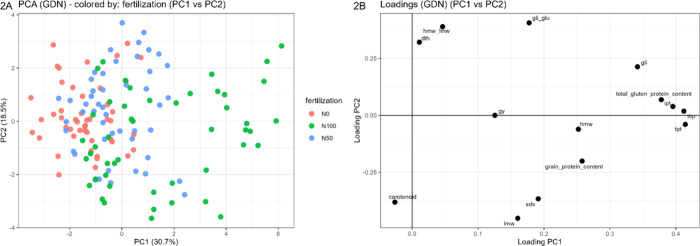
PCA of the GDN trial data. (A) PC1–PC2 score plot, where samples are colored according to nitrogen fertilization level: N0 = control fertilization; N50 = 50 units of nitrogen fertilization; N100 = 100 units of nitrogen fertilization. (B) PC1–PC2 loading plot, showing the contribution of each variable distributed on PC1 and PC2, where gy = grain yield (t/ha); dth = days to heading; carotenoid (g/100 g of flour); sds = sodium dodecyl sulfate sedimentation assay; protein content (%, NIR); ipt and tpt = immunogenic and toxic peptides (ppm); TITP = total immunogenic and toxic peptides (ppm), gli, lmw, and hmw = glutenin fractions (mg/g flour); gli_glu and hmw_lmw = gluten quality indices (gli/glu, hmw-lmw); total gluten protein content = gli + lmw + hmw (mg/g flour).

### GSO Trial

The analysis of variance of the GSO trial showed a significant result only in the main three factors, as reported in the Supporting Information, Tables S7 and S8. No significant interactions among factors were detected for the protein-related traits; the only significant interactions were observed for grain yield and carotenoids. Therefore, in the following sections, only results for the main independent variables are presented.

#### Genotype

The two durum wheat genotypes Cannizzo and Saragolla exhibited distinct differences in yield and grain quality traits, as reported in Figure 3. Although no statistically significant difference was observed in TPT, notable variations emerged in IPT (1617–1354 ppm), protein content (14.57–13.49 g/100 g), and grain yield (2.77–2.94 t/ha). Cannizzo accumulated 17.7% more IPT compared to Saragolla. These results suggest a potential trade-off between grain quality (in terms of protein quantity and immunogenic load) and overall productivity, influenced by genotype.

**Figure 3:**
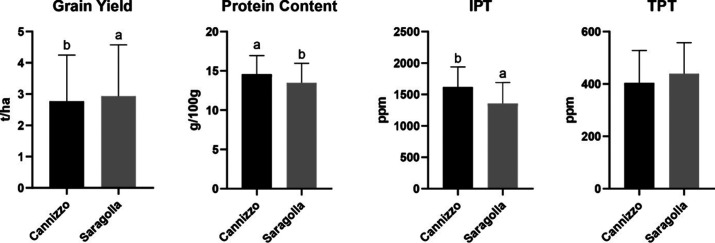
Effect of genotype (Cannizzo and Saragolla) on grain yield, protein content, and IPT and TPT epitopes values. Different letters indicate statistically significant differences between genotypes (*p* < 0.05, ANOVA followed by Duncan post hoc test). No letters mean no statistically significant differences.

#### Sowing Date

Sowing dates influenced productivity and protein-related traits, as reported in Figure 4. Fall sowing strongly influenced the grain yield (4.35 t ha^–1^) compared to spring yield (1.36 t ha^–1^), with a decrease of 100% in the latter. On the other hand, in the fall-planted wheats, there was a reduction of 27.16% in total protein content (11.83 g/100 g for fall and 16.24 g/100 g for spring) and 22.71% IPT levels (1334 and 1637 ppm for fall and spring sowing, respectively). For the TPT, no significant differences were observed. These findings suggest that spring sowing may trigger the accumulation of stress-related proteins, including those with immunogenic or technological relevance, with a negative impact on productivity.

**Figure 4:**
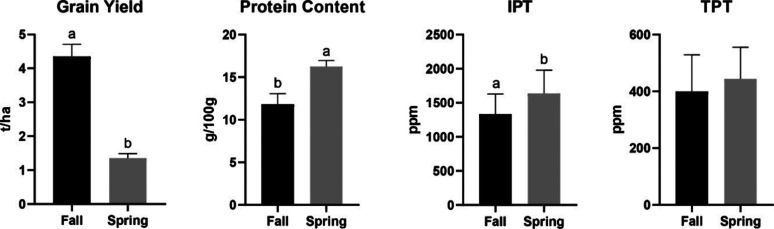
Effects of sowing date (fall and spring sowing) on grain yield, protein content, and IPT and TPT epitopes values. Different letters indicate statistically significant differences between genotypes (*p* < 0.05, ANOVA followed by Duncan *post hoc* test). No letters mean no statistically significant differences.

##### Fertilization

Nitrogen fertilization significantly influenced yield, protein content, IPT, and TPT, indicating its impact on grain protein composition (Figure 5). Among the three treatments, mineral N led to the highest values across all measured parameters and increased the IPT of 29.07% and TPT of 49.85% compared to the control. On the other hand, organo-mineral (Nutrigrantop) N fertilization achieved a grain yield comparable to that obtained with mineral fertilization (2.93–2.99 t/ha) and a lower IPT accumulation of 6.27% compared to mineral N fertilization. For the TPT, no differences were observed; the parts per million value was intermediate between the control and mineral fertilization. These results underscore the role of nitrogen fertilization not only in enhancing productivity but also in modulating protein synthesis, including protein fractions with potential technological and immunogenic significance.

**Figure 5:**
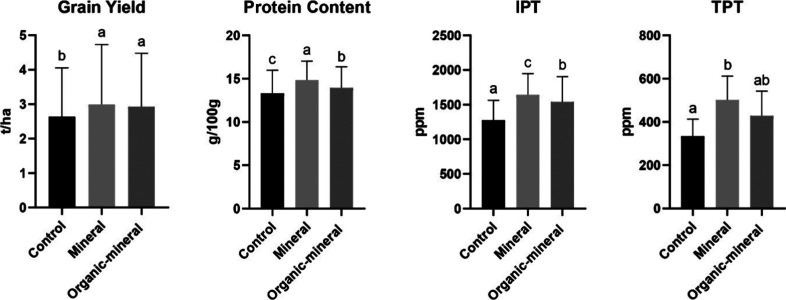
Effect of fertilization (Control = 0, Mineral = N–P 18–46, Organic-mineral 12 25 Nutrigrantop S) on grain yield, protein content, IPT, and TPT epitope values. Different letters indicate statistically significant differences between genotypes (*p* < 0.05, ANOVA followed by Duncan *post hoc* test).

#### Multivariate Structure of Yield and Quality Traits in the GSO Trial: Pearson Correlations and PCA

The Pearson correlation matrix revealed a structured covariance pattern among phenology, yield, and grain quality traits in the GSO trial (Figure S11). The immunogenicity indices were strongly and positively intercorrelated (IPT vs TPT, *r* = 0.75; TITP vs IPT, *r* = 0.98; TITP vs TPT, *r* = 0.85), indicating that these variables capture a shared immunogenicity-related dimension. In contrast to GDN, immunogenicity indices showed negative associations with gluten composition traits, including gliadins (TITP vs gli, *r* = −0.43; TPT vs gli, *r* = −0.44; IPT vs gli, *r* = −0.40) and total gluten protein content (TITP vs total gluten protein content, *r* = −0.25; TPT vs total gluten protein content, *r* = −0.32; IPT vs total gluten protein content, *r* = −0.21). Gluten composition traits remained highly coordinated (total gluten protein content versus gli, *r* = 0.94). Grain yield and days to heading were nearly perfectly correlated (gy vs dth, *r* = 0.99), and both were strongly and negatively associated with grain protein content surrogate (dth vs total gluten protein content, *r* = −0.92; gy vs grain protein content, *r* = −0.88). Overall, the presence of correlated trait blocks and opposing trait sets supported the application of PCA as a multivariate synthesis of the covariation structure.

In the PCA, the scree plot indicated that variance was distributed across multiple components (Figure S12), with PC1 explaining 42.2%, PC2 19.6%, and PC3 15.1% of the total variability (cumulative 76.9%). The PC1–PC2 loading plot (Figure 6A) and the PC1–PC2 score plot of the sowing date (Figure 6B) capture the largest share of variance in a two-dimensional representation (61.8%) and provide the clearest visualization of the dominant management gradient. PC1 primarily contrasted the immunogenicity variable (TITP, IPT, and TPT; positive loadings) together with grain protein content versus gliadins and total gluten protein content (negative loadings), consistent with the correlation structure (Figure 6A). PC2 mainly reflected variation associated with phenology and yield, with days to heading and grain yield loading positively. In the score plot (Figure 6B), samples were clearly separated by sowing date, indicating that fall versus spring sowing was associated with a marked shift in the multivariate trait profile along the primary PCA axes.

**Figure 6:**
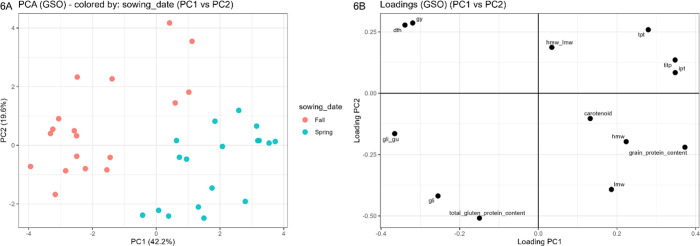
PCA of the GSO trial data. (A) PC1–PC2 score plot, where samples are colored according to sowing date: Fall = fall sowing (12/12/2016); Spring = spring sowing (22/02/2017). (B) PC1–PC2 loading plot, showing the contribution of each variable distributed on PC1 and PC2, where gy = grain yield (t/ha); dth = days to heading; carotenoid (g/100 g of flour); sds = sodium dodecyl sulfate sedimentation assay; protein content (%, NIR); ipt and tpt = immunogenic and toxic peptides (ppm); titp = total immunogenic and toxic peptides (ppm); gli, lmw, and hmw = glutenin fractions (mg/g flour); gli_glu and hmw_lmw = gluten quality indices (gli/glu, hmw-lmw); total gluten protein content = gli + lmw + hmw (mg/g flour).

A less marked but significant effect of genotypes was also observed along PC3, as reported in the relevant PC1–PC3 score plot (Figure S17). The corresponding PC1–PC3 loading plot (Figure S12) shows that PC3 is mainly influenced by HMW/LMW and carotenoids at positive values, and by LMW at negative values, which suggests that the systematic differences between the two genotypes are mainly related to these quantitative traits.

## Discussion

Gluten-related disorders affect a significant portion of the population, yet not everyone with gluten sensitivity is diagnosed with celiac disease.^37^ Given that durum wheat and other gluten-containing cereals are not safe for people with celiac disease, understanding the variability of gluten peptides across different wheat types and growing conditions may help improve strategies for managing gluten exposure. This kind of research is useful to understand how environmental factors shape gluten’s composition, which is crucial for breeding wheat varieties with potentially lower immunogenicity.^17,38,39^ Ultimately, the research outputs can improve wheat quality, guide dietary advice for people with gluten sensitivities, and support the development of wheat products that are safer for a broader range of consumers.^40,41^ Furthermore, it is an important step toward balancing the nutritional and cultural value of wheat with the growing need for gluten-aware food options.

The notion that the genetic background of a wheat variety is the primary driver of its gluten protein profile, as highlighted by the present study, has been known for a long time. This finding aligns with research from Ronga and colleagues,^17^ showing that gluten composition and immunogenic peptides are accumulated in different proportions even in modern breeding varieties. In comparison, Boukid et al.^42^ observed differences among landraces, as well as old and modern wheat with no specific trend associating allergenicity to modern breeding programs; our results show that the Cannizzo genotype, for instance, had significantly higher levels of immunogenic peptides (IPT) compared to Saragolla. This supports the idea that inherent genetic differences control the synthesis of gluten proteins, including those with a higher potential for triggering an immune response. Beyond the influence of the genotype, our study highlights the powerful influence of agronomic factors. The sowing date significantly altered the peptide profile; fall-sown wheat had notably lower protein and IPT levels than spring-sown wheat, independently of the genotype.

This is likely due to the cooler temperatures and more favorable moisture conditions during the vegetative phase in the fall sowing. This observation is consistent with other research showing that climatic stress, such as high temperatures and water deficits, can dramatically affect gluten composition.^16,42^ Graziano et al.^16^ reported that high temperature and low rainfall during grain filling differently affected gluten composition and immunogenic peptide levels in the old Cappelli and modern Saragolla cultivars, indicating a genotype-dependent response to environmental stress. In contrast, Boukid and colleagues^42^ showed that severe rainfed conditions during grain filling altered peptide profiles, suggesting that water deficit can trigger convergent protein accumulation mechanisms irrespective of genetic background. Therefore, environmental modulation through the sowing date should be viewed as a complementary strategy, best coupled with breeding programs targeting inherently low-immunogenic wheat cultivars.

A critical finding across our trials is the inverse relationship between productivity and peptide accumulation, often termed the dilution effect. Generally, genotypes that are less productive tend to accumulate more total protein and, consequently, higher concentrations of immunogenic and toxic peptides (IPT and TPT).^16,42^ This pattern was confirmed under conditions that constrained yield, such as delayed spring sowing (GSO trial) and the use of excessive plant density (e.g., 400 plants/m^2^ in the GDN trial). Conversely, high grain yields, whether achieved through optimal genotype performance or appropriate fall sowing, contribute to a dilution of the protein and epitope contents within the kernel. This suggests that agronomic practices promoting the maximum yield potential are also beneficial for reducing the immunogenic load.

Similarly, our results reveal a clear trade-off associated with nitrogen fertilization. We found that increased mineral nitrogen inputs boosted total protein content but also led to a significant increase in both IPT and TPT. This is consistent with the well-established link between N availability and gliadin synthesis.^22,43–46^ This raises the challenge of balancing nutritional and technological quality with potential health risks for sensitive populations. The observed moderating effect of organic–mineral fertilization suggests that integrated nutrient management could partially decouple protein content from IPT, offering a pathway toward improved protein quality without exacerbating allergenic potential. However, such strategies must be evaluated in the context of long-term yield, environmental sustainability, and economic viability for farmers.

Furthermore, our results suggest a difference in the sensitivity of the two peptide groups to management factors. In general, the IPT appeared to be more sensitive and broadly responsive to the agronomic factors analyzed (genotype, sowing density, and fertilization levels). TPT, while influenced, seems to be more strongly influenced by fertilization management, particularly mineral fertilization. This heightened sensitivity of TPT to mineral inputs is due to the fact that mineral N (especially from N–P 18–46 and N100) is readily available to the plant, driving protein biosynthesis during the grain filling stage. The accumulation of gliadins, which contain most of the TPT epitopes, is strictly correlated with N availability.^43,45^ Consequently, TPT appears to be modulated more directly by the immediate availability of N compared to other factors, which exert a more indirect influence through yield.^16,46^

The observed interaction effects between genotype, sowing density, and fertilization expose the inherent complexity in managing immunogenic peptide levels through agronomic means.^17^ Our GDN trial showed that the favorable outcomes, balancing high yield with low IPT and TPT levels, were observed through specific combinations of these factors. For example, Saragolla at a higher sowing density with moderate nitrogen (N50) proved to be a potentially favorable combination. This complexity highlights a critical challenge: There is no one-size-fits-all agronomic solution. Effective management strategies must be highly specific, and efforts to mitigate immunogenicity risk require a multidisciplinary approach that integrates molecular breeding with precision agronomic protocols.^47,48^ Without this precision, efforts to mitigate the immunogenicity risk may remain fragmented and inconsistent.

Southern Italy represents a strategic area for durum wheat cultivation. However, in Mediterranean environments, where rainfall is mainly concentrated in fall and winter and prolonged drought periods frequently occur during spring and summer, it is essential to define appropriate crop management strategies to achieve sufficient grain yield and quality while at the same time minimizing the content of peptides associated with the development of celiac disease. In line with previous studies reporting that both genotype choice and agronomic management can significantly influence the accumulation of immunogenic gluten peptides, the results obtained in this study are promising and provide a preliminary step in this direction. It would also be of great interest to extend this investigation to a wider combination of years and environments, to better capture genotype × environment × management interactions and to confirm the robustness of the observed trends. Even if important advancements have been obtained, this study still presents some constraints, since it was conducted with a limited number of genotypes over a single growing season in one location, which restricts the generalizability of the findings. Future research should expand the scope to include a wider range of genotypes and multienvironment trials. Particular attention should be given to identifying stable cultivars capable of combining a satisfactory grain yield with consistently low IPT and TPT levels across contrasting environments and management conditions. Furthermore, to provide a complete picture, future studies should integrate other important end-use quality traits, such as dough rheology and baking/pasta performance, to ensure that strategies for reducing immunogenicity do not compromise the functional quality of the final product. Ultimately, this research provides a starting point for developing comprehensive breeding and management strategies that can balance productivity, health considerations, and end-use quality.

The present study indicates that agronomic practices, specifically genotype selection, sowing date, and fertilization management, serve as initial tools for modulating the balance between durum wheat yield and grain quality, particularly with respect to the accumulation of immunogenic and toxic peptides involved in the celiac disease.

While this research was not aimed at creating a gluten-free product, our findings demonstrate that genotype, sowing date, and fertilization management strongly influence the accumulation of immunogenic and toxic peptides in durum wheat. Genetic background remains the primary determinant of peptide composition, yet environmental modulation, as sowing date and nutrient management strategies can significantly alter the expression of these traits. The observed trade-offs between protein content, yield, and peptide levels, especially the significant genotype by agronomic management practices interaction, prove that a ″one-size-fits-all″ agronomic solution is ineffective. Effective management requires a precise, genotype-specific approach to balance productivity with the goal of producing grain with reduced immunogenic potential.

These findings open new scenarios for developing new cultivation strategies to produce wheat with lower levels of toxic peptides, making it a potentially more suitable option for individuals with gluten sensitivities without eliminating the benefits of wheat in the diet.

## Supplementary Material





## Notes

(Figure S1) (a) line graph of mean temperatures (°C) of the 2016/2017 cropping season compared with the mean long temperature of the period ranging from 1953 to 2012, and (b) bar graph of rainfall accumulation (mm) of the 2016/2017 cropping season compared to the rainfall accumulation of the period ranging from 1953 to 2012; (Figure S2) combined graph of minimum and maximum daily temperatures and rainfall accumulation for the 2016/2017 cropping season; (Figure S3) bar plot showing total radiation throughout the 2016/2017 cropping season; (Figure S4) pairwise Pearson’s correlation coefficients (*r*) among agronomic, phenological, and grain quality traits in the GDN trial; (Figure S5) PCA loading plot of PC1–PC3 of the GDN trial; (Figure S6) PCA score plot for the GDN trial (PC1 vs PC3) by nitrogen fertilization; (Figure S7) PCA score plot for the GDN trial (PC1 vs PC2) by sowing density; (Figure S8) PCA score plot for the GDN trial (PC1 vs PC3) by sowing density; (Figure S9) PCA score plot for the GDN trial (PC1 vs PC2) by genotype; (Figure S10) PCA score plot for the GDN trial (PC1 vs PC3) by genotype; (Figure S11) Pairwise Pearson’s correlation coefficients (*r*) among agronomic, phenological, and grain quality traits in the GSO trial; (Figure S12) PCA loading plot of PC1–PC3 of the GSO trial; (Figure S13) PCA score plot for the GSO trial (PC1 vs PC3) by sowing date; (Figure S14) PCA score plot for the GSO trial (PC1 vs PC2) by fertilization; (Figure S15) PCA score plot for the GSO trial (PC1 vs PC3) by fertilization; (Figure S16) PCA score plot for the GSO trial (PC1 vs PC2) by genotype; and (Figure S17) PCA score plot for the GSO trial (PC1 vs PC3) by genotype (PDF)
(Table S1) Skeleton ANOVA tables for the GDN trial—eight Genotypes, sowing Density and Nitrogen fertilization trial performed to test the influence of agronomic practices on durum wheat grain quality; (Table S2) main agronomic traits and grain quality of durum wheat cultivars; (Table S3) skeleton three-way ANOVA tables for the GSO trial—two Genotypes, Sowing date and Organic-mineral fertilization trial performed to test the influence of agronomic practices on durum wheat grain quality; (Table S4) Nutrigrantop S composition; (Table S5) significance levels of the main effects and interactions from the ANOVA for yield and quality traits for GDN trial; (Table S6) result of the main effects and interactions from the ANOVA analysis for yield and quality traits GDN trial; (Table S7) significance levels of the main effects and interactions from the ANOVA analysis for yield and quality traits for GSO trial; (Table S8) result of the main effects and interactions from the ANOVA analysis for yield and quality traits GSO trial (XLSX)

## †

Present Address: ClonBio Group Ltd., 6 Fitzwilliam Pl, D02 XE61 Dublin, Ireland

## Abbreviations

HMW-GS: high-molecular-weight glutenin subunits
LMW-GS: low-molecular-weight glutenin subunits
CD: celiac disease
TPT: toxic epitopes
IPT: immunogenic epitopes
TITP: total immunogenic and toxic epitopes
G × E: Genotype × Environment
GDN: Genotype, sowing Density, and Nitrogen fertilization trial
GSO: Genotypes Sowing date and Organic-fertilization trial
RCBD: randomized complete block design
GS: Zadoks growth stages
MCPA: 2-methyl-4-chlorophenoxyacetic acid
DtH: days to heading
GY: grain yield
GPC: grain protein content
SDS: sodium dodecyl sulfate sedimentation assay
RP-UPLC/ESI-MS: reverse-phase ultraperformance liquid chromatography coupled with electrospray ionization mass spectrometry
GS: glutenin subunits
DTT: dithiothreitol
ACN: acetonitrile
TFA: trifluoroacetic acid
ANOVA: analysis of variance
PCA: principal component analysis
PC1: first principal component
PC2: second principal component
Gli-Glu: gliadin-to-glutenin ratio.
