# Supplementary material for: Genotype and Agronomic Management Interactions Shape the Accumulation of Immunogenic and Toxic Gluten Peptides in Durum Wheat
Source: J Agric Food Chem. 2026 Apr 28;74(17):13505–15. doi: 10.1021/acs.jafc.5c16192 (PMC13154179; doi:10.1021/acs.jafc.5c16192)

Title: Genotype and Agronomic Management Interactions Shape the Accumulation of Immunogenic and Toxic Gluten Peptides in Durum Wheat

Authors: Giovanni Caccialupi[1*‡], Leonardo Cicala[1*‡], Justyna Milc[1], Alessandro Ulrici[1], Fatma Boukid[2,†], Arnaldo Dossena[2], Sara Graziano[2], Barbara Prandi[2], Giovanna Visioli[2], Nelson Marmiroli[2], Mariolina Gullì[2], Pasquale De Vita[3], Nicola Pecchioni[1,3], Enrico Francia[1]

Affiliations:

[1] Department of Life Sciences, University of Modena and Reggio Emilia, Via Amendola 2, Pad. Besta, 42122 Reggio Emilia, Italy;

[2] Department of Chemistry, Life Sciences and Environmental Sustainability, Centre SITEIA. PARMA, University of Parma, 43124 Parma, Italy

[3] Research Centre for Cereal and Industrial Crops, CREA-CI, 71122 Foggia, Italy;

Corresponding Author: *Giovanni Caccialupi; giovanni.caccialupi@unimore.it

‡Giovanni Caccialupi and Leonardo Cicala contributed equally.

Figure S1. Figure 1. (a) Line graph of mean temperature (°C); dotted line shows the mean temperature per month of 2016/2017 cropping season; continuous line shows the mean long temperature of the period ranging from 1953 to 2012. –(b) Bar graph of rainfall accumulation (mm); dotted bars show the rainfall accumulation per month of 2016/2017 cropping season; full bars show the rainfall accumulation of the period ranging from 1953 to 2012.

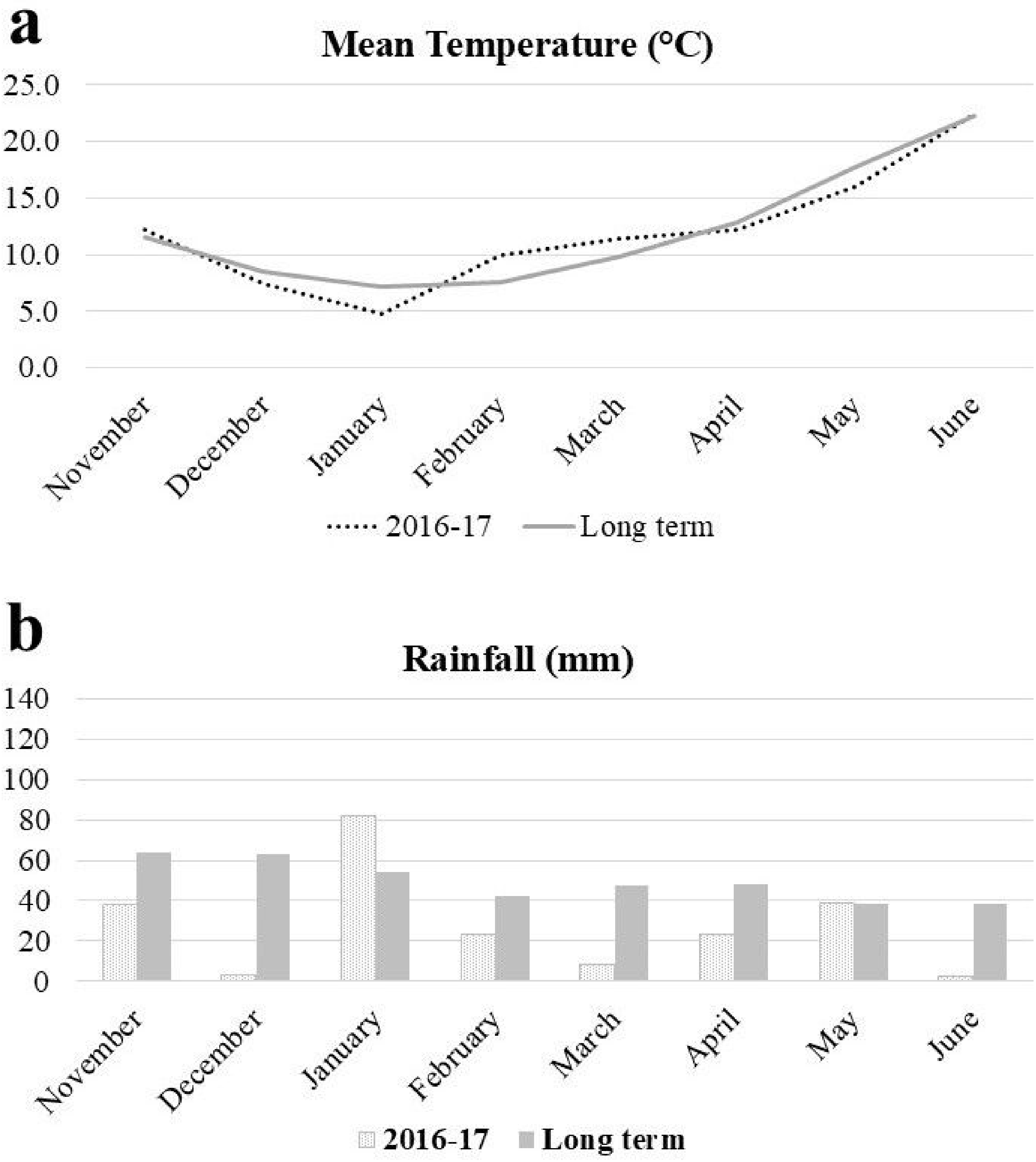

Figure S2. Combined graph of rainfall accumulation (mm), minimum and maximum temperature (°C) and rainfall accumulation (mm) of the cropping season 2016/2017.

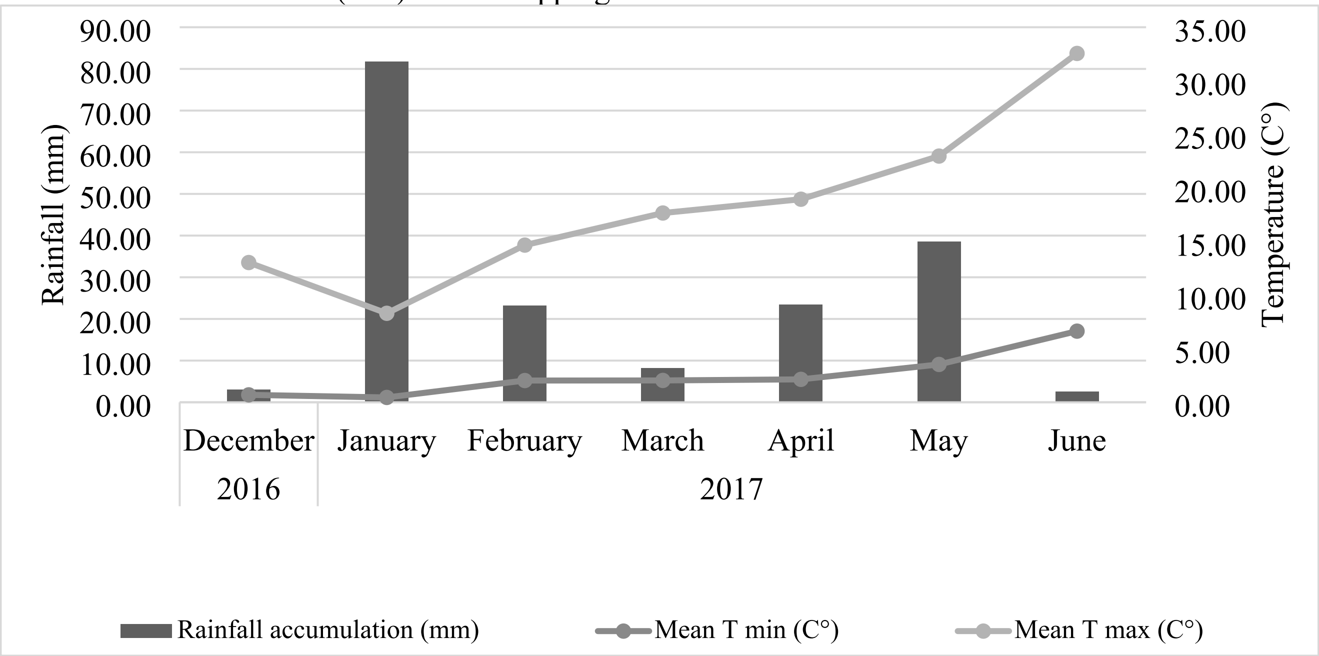

Figure S3. Bar plot of the total solar radiation during the experimental period at the CREA meteorological station (Foggia, Italy). Monthly means daily values of total (global) solar radiation are shown from December 2016 to June 2017. Data were recorded by the CREA weather station and are reported as monthly averages of daily totals (MJ/m^2d^1).

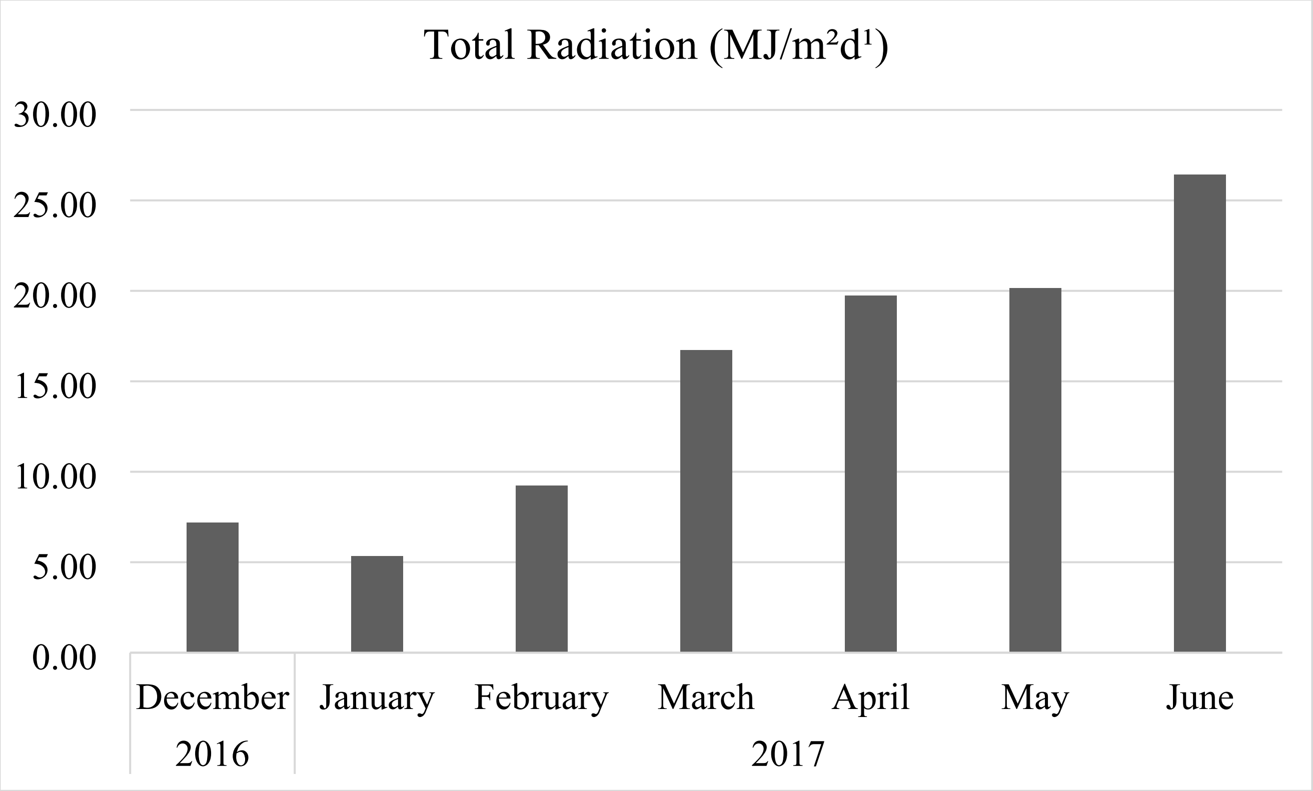

Figure S4. Pairwise Pearson's correlation coefficients (*r*) among agronomic, phenological, and grain quality traits in the GDN trial. Abbreviations: gy, grain yield (t/ha); dth, days to heading; carotenoid (g/100g of flour), sds, Sodium Dodecyl Sulfate sedimentation assay, protein content (%, NIR), immunogenic and toxic peptides (IPT ppm, TPT ppm), total immunogenic and toxic peptides (TITP ppm), glutenin fractions (GLI mg/g flour, LMW mg/g flour, HMW mg/g flour flour), gluten quality indices (Gli/Glu, Hmw-Lmw) Total Gluten Protein Content (Gli + LMW + HMW; mg/g flour).

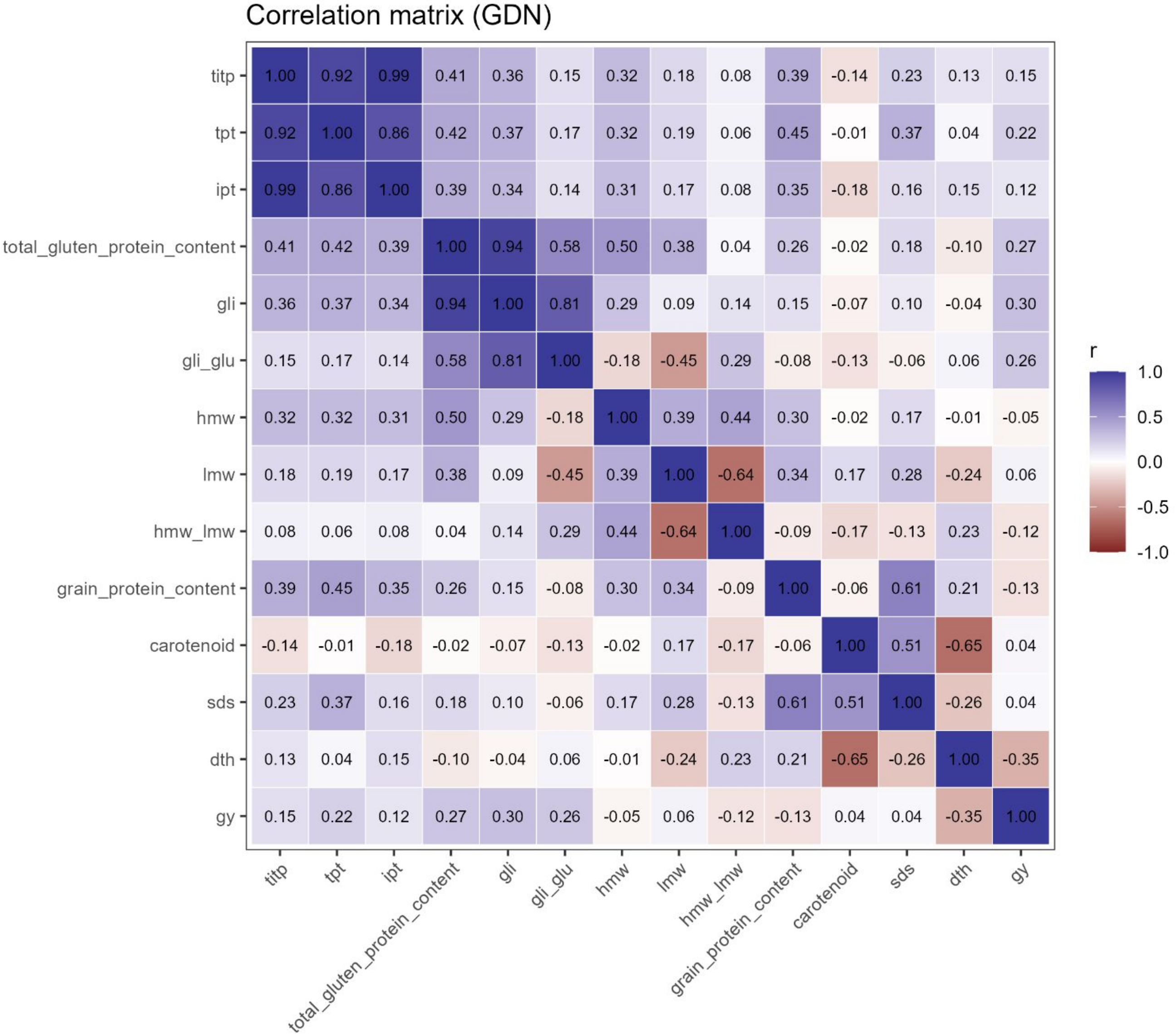

Figure S5. PCA loading plot of PC1-PC3 of the GDN trial. gy, grain yield (t/ha); dth, days to heading; carotenoid (g/100g of flour), sds, Sodium Dodecyl Sulfate sedimentation assay, protein content (%, NIR), immunogenic and toxic peptides (IPT ppm, TPT ppm), total immunogenic and toxic peptides (TITP ppm), glutenin fractions (GLI mg/g flour, LMW mg/g flour, HMW mg/g flour flour), gluten quality indices (Gli/Glu, Hmw-Lmw) Total Gluten Protein Content (Gli + LMW + HMW; mg/g flour).

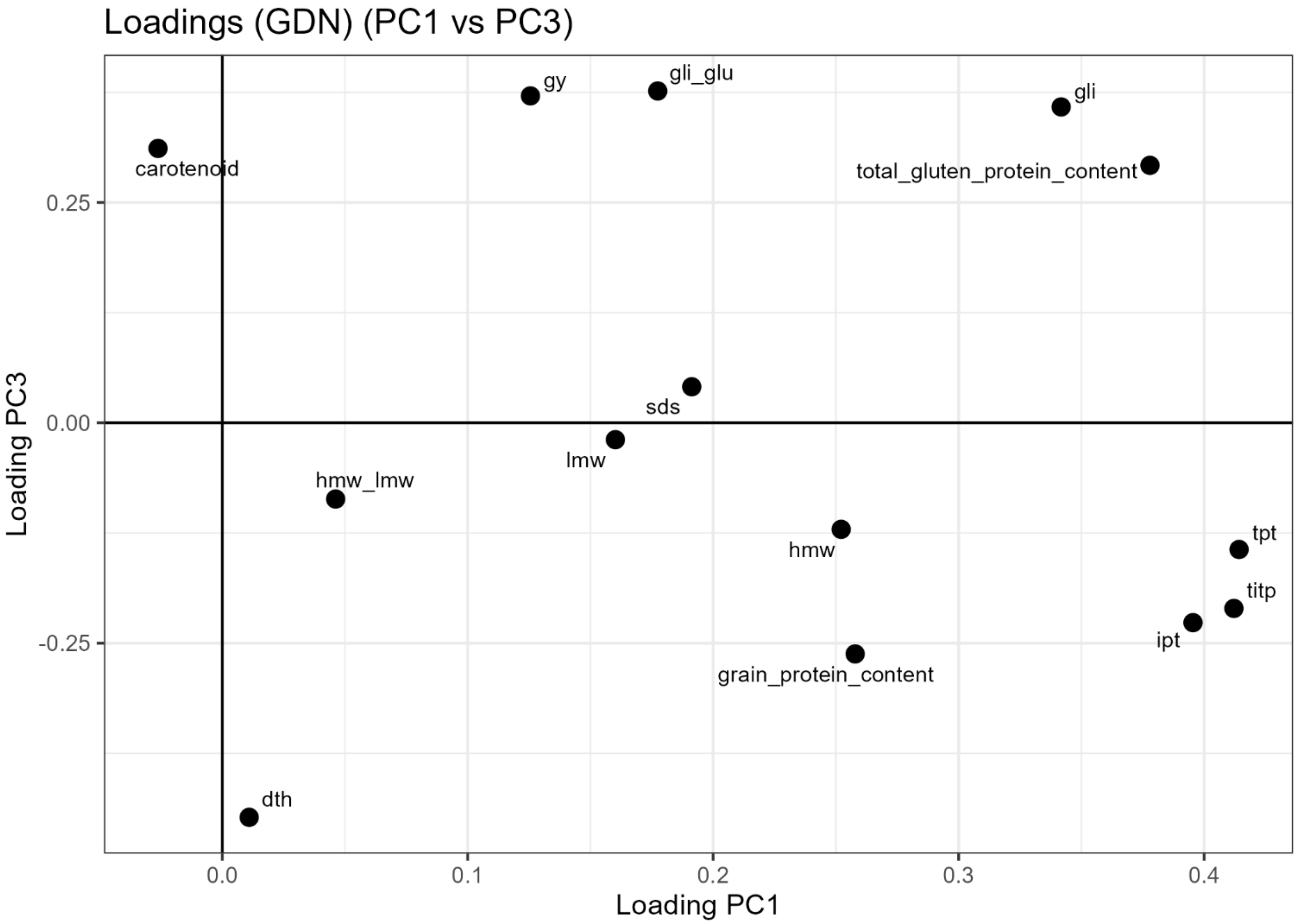

Figure S6. PCA score plot for the GDN trial (PC1 vs PC3) by nitrogen fertilization. Scores of plot-level observations projected on PC1 (30.7%) and PC3 (15.2%) from PCA on standardized response variables. Points are colored by N level (red, N0; N50, blue; N100, green).

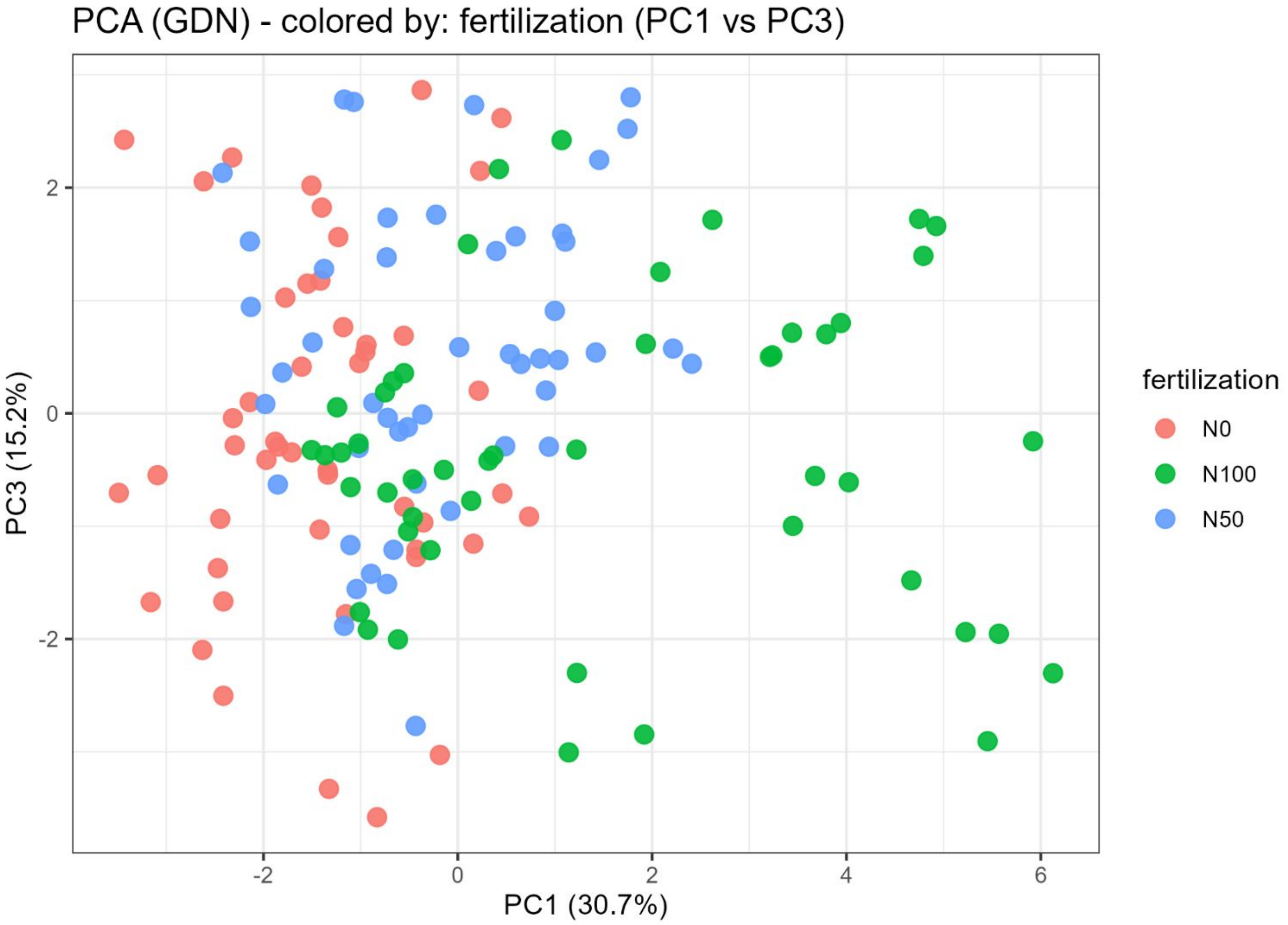

Figure S7. PCA score plot for the GDN trial (PC1 vs PC2) by sowing density. Scores of plot-level observations projected on PC1 (30.7%) and PC2 (18.5%) from PCA on standardized response variables. Points are colored by sowing density (red, 200; light blue, 400).

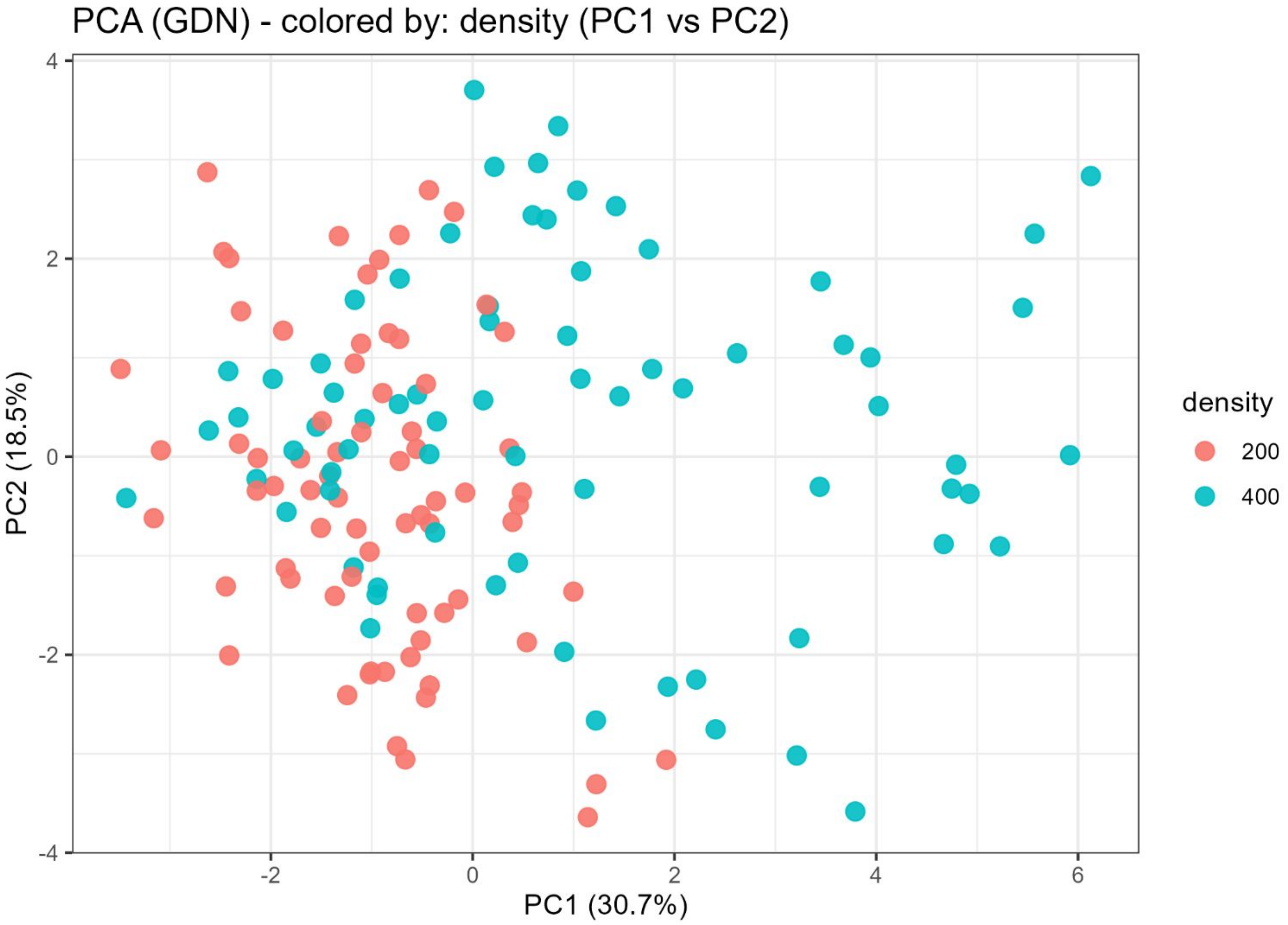

Figure S8. PCA score plot for the GDN trial (PC1 vs PC3) by sowing density. Scores of plot-level observations projected on PC1 (30.7%) and PC3 (15.2%) from PCA on standardized response variables. Points are colored by sowing density (red, 200; light blue, 400).

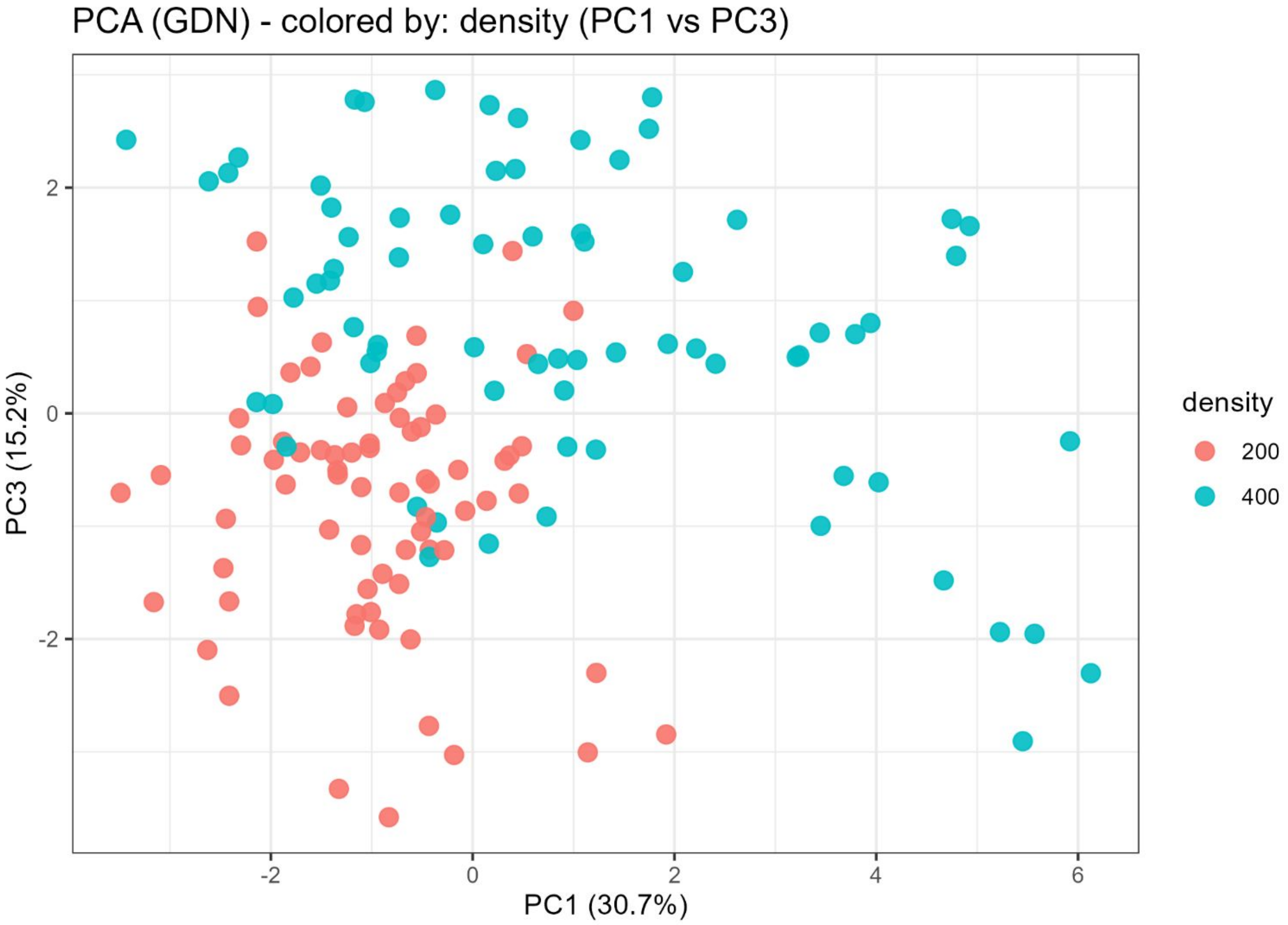

Figure S9. PCA score plot for the GDN trial (PC1 vs PC2) by genotype. Scores of plot-level observations projected on PC1 (30.7%) and PC2 (18.5%) from PCA on standardized response variables. Points are colored by genotype (red, Aureo; yellow, Cannizzo; light green, Creso; light blue, Saragolla; blue, Senatore Cappelli, violet, Simeto; pink, Svevo).

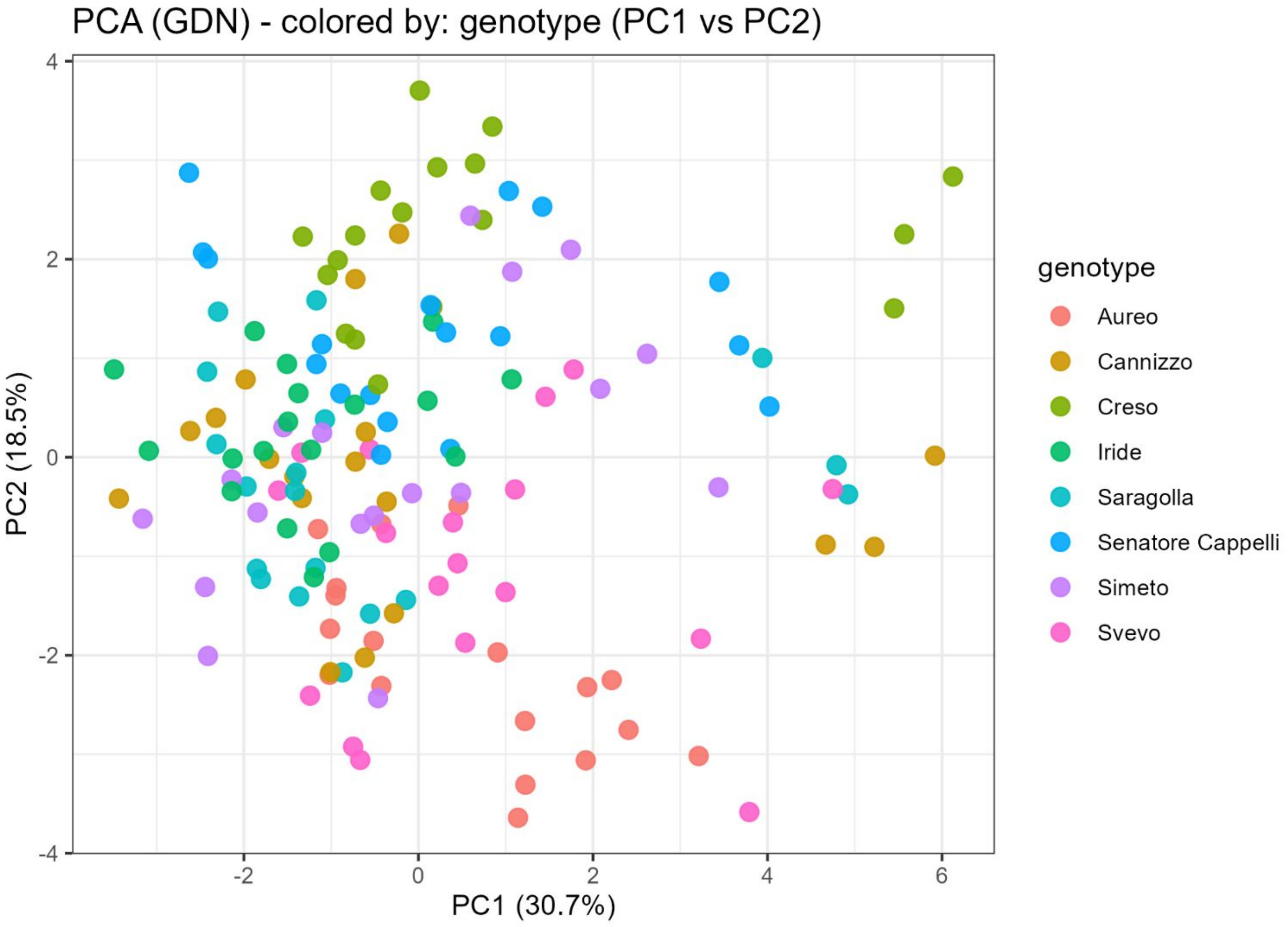

Figure S10. PCA score plot for the GDN trial (PC1 vs PC3) by genotype. Scores of plot-level observations projected on PC1 (30.7%) and PC3 (15.2%) from PCA on standardized response variables. Points are colored by genotype (red, Aureo; yellow, Cannizzo; light green, Creso; light blue, Saragolla; blue, Senatore Cappelli, violet, Simeto; pink, Svevo).

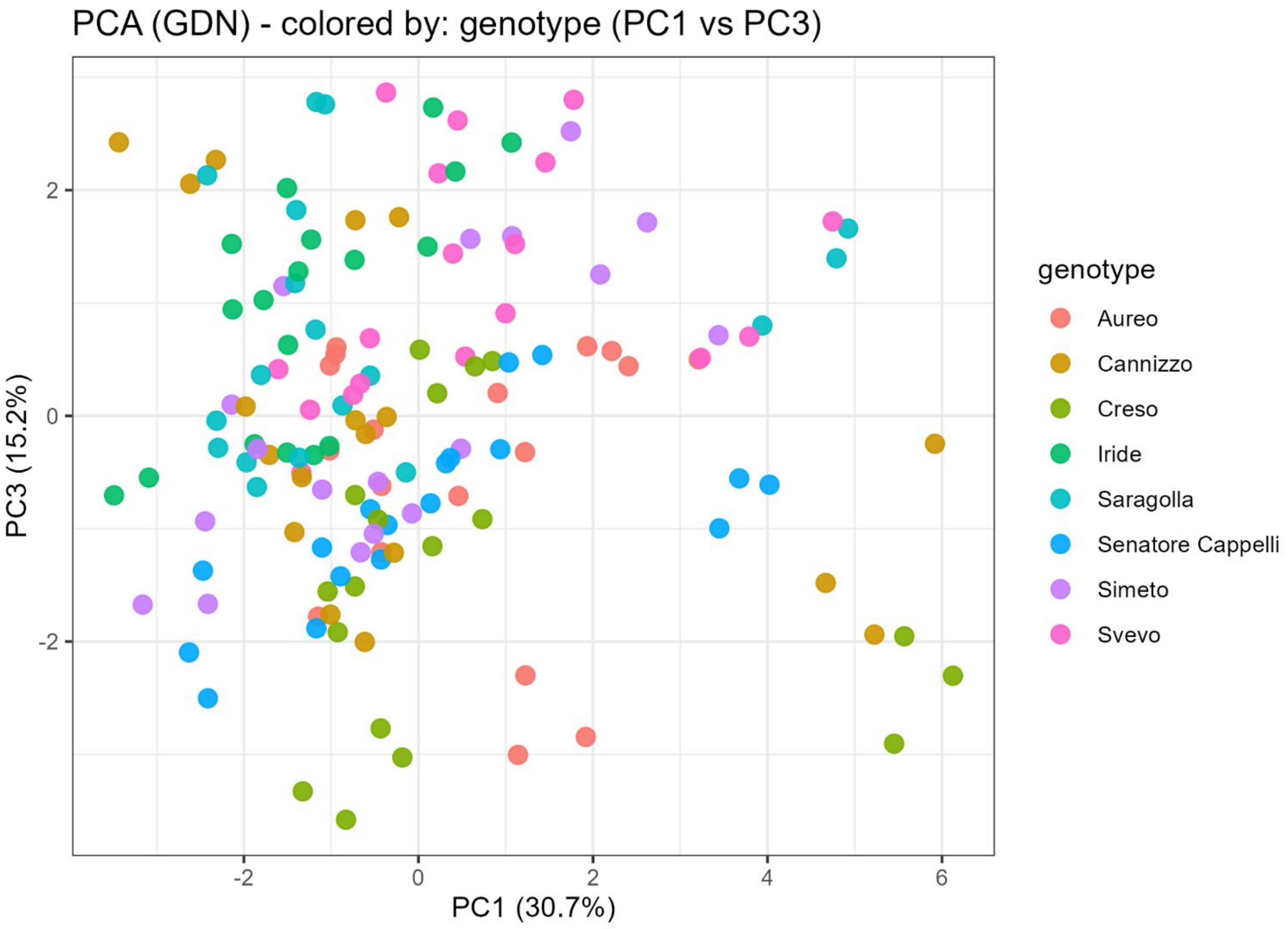

Figure S11. Pairwise Pearson's correlation coefficients (*r*) among agronomic, phenological, and grain quality traits in the GSO trial Abbreviations: gy, grain yield (t/ha); dth, days to heading; carotenoid (g/100g of flour), sds, Sodium Dodecyl Sulfate sedimentation assay, protein content (%, NIR), immunogenic and toxic peptides (IPT ppm, TPT ppm), total immunogenic and toxic peptides (TITP ppm), glutenin fractions (GLI mg/g flour, LMW mg/g flour, HMW mg/g flour flour), gluten quality indices (Gli/Glu, Hmw-Lmw) Total Gluten Protein Content (Gli + LMW + HMW; mg/g flour).

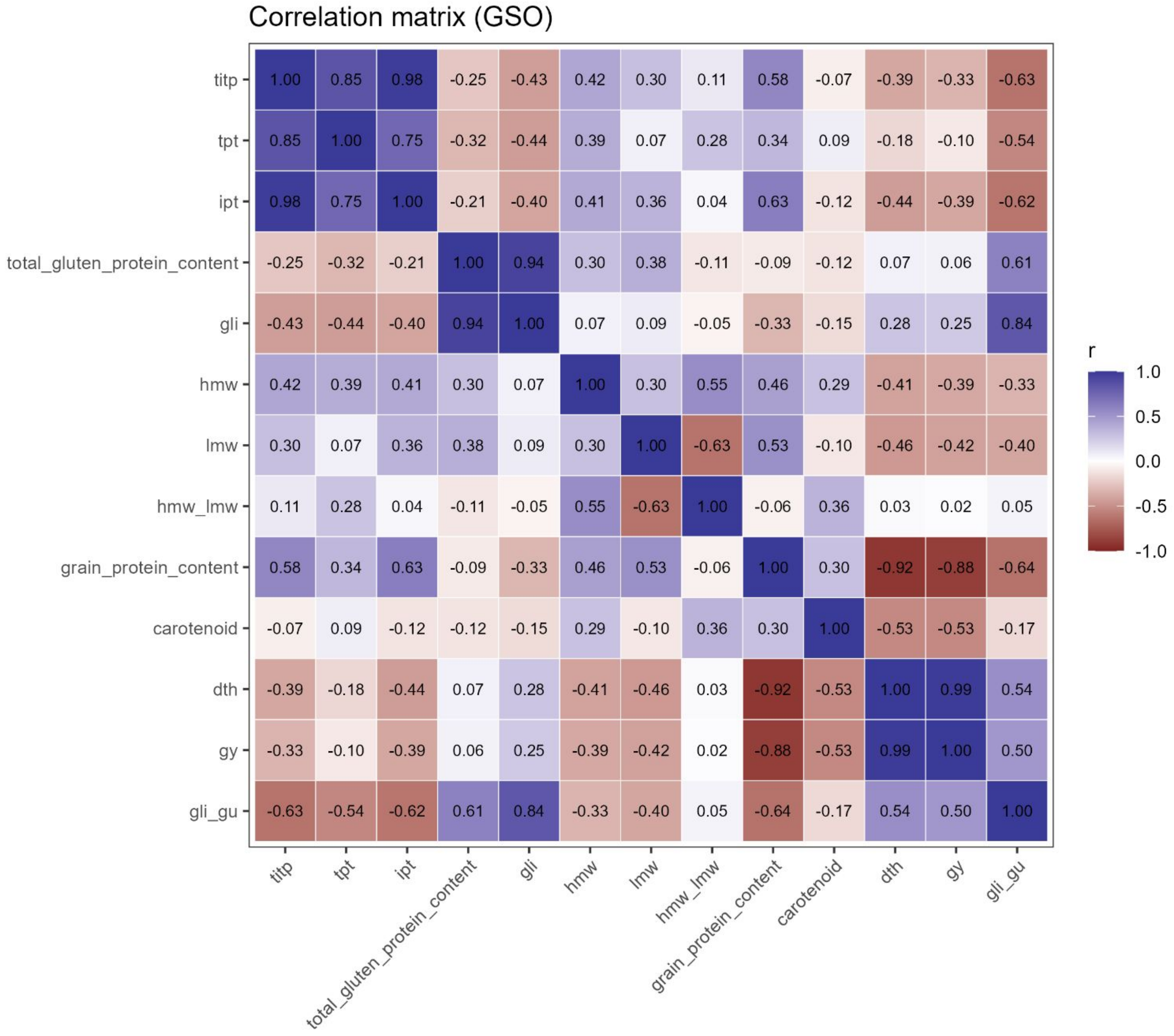

Figure S12. PCA loading plot of PC1-PC3 of the GSO trial. gy, grain yield (t/ha); dth, days to heading; carotenoid (g/100g of flour), protein content (%, NIR), immunogenic and toxic peptides (IPT ppm, TPT ppm), total immunogenic and toxic peptides (TITP ppm), glutenin fractions (GLI mg/g flour, LMW mg/g flour, HMW mg/g flour flour), gluten quality indices (Gli/Glu, Hmw-Lmw) Total Gluten Protein Content (Gli + LMW + HMW; mg/g flour).

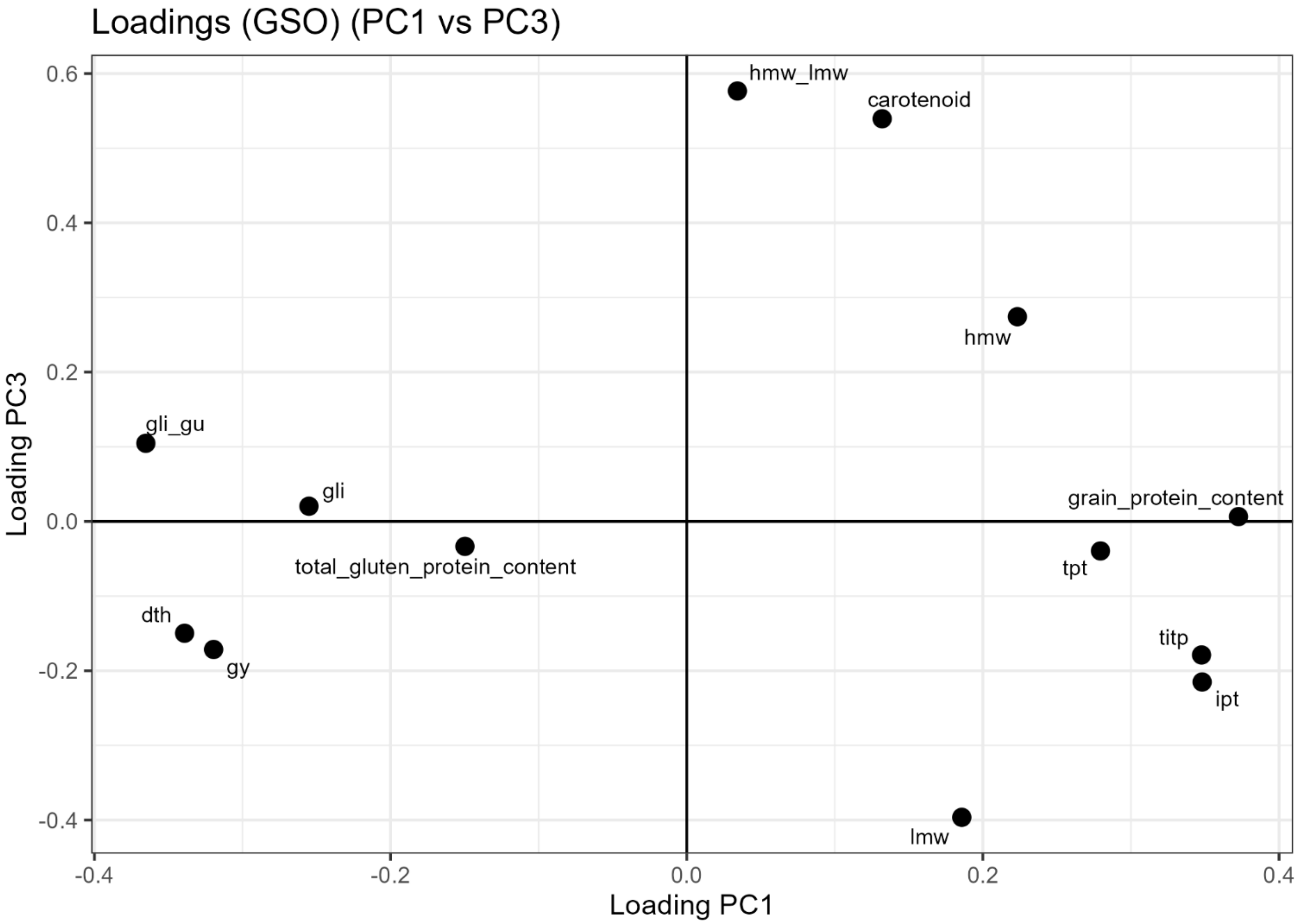

Figure S13. PCA score plot for the GSO trial (PC1 vs PC3) by sowing date. Scores of plot-level observations projected on PC1 (42.2%) and PC3 (15.1%) from PCA on standardized response variables. Points are colored by sowing date (red, fall-sowing; blue, spring sowing).

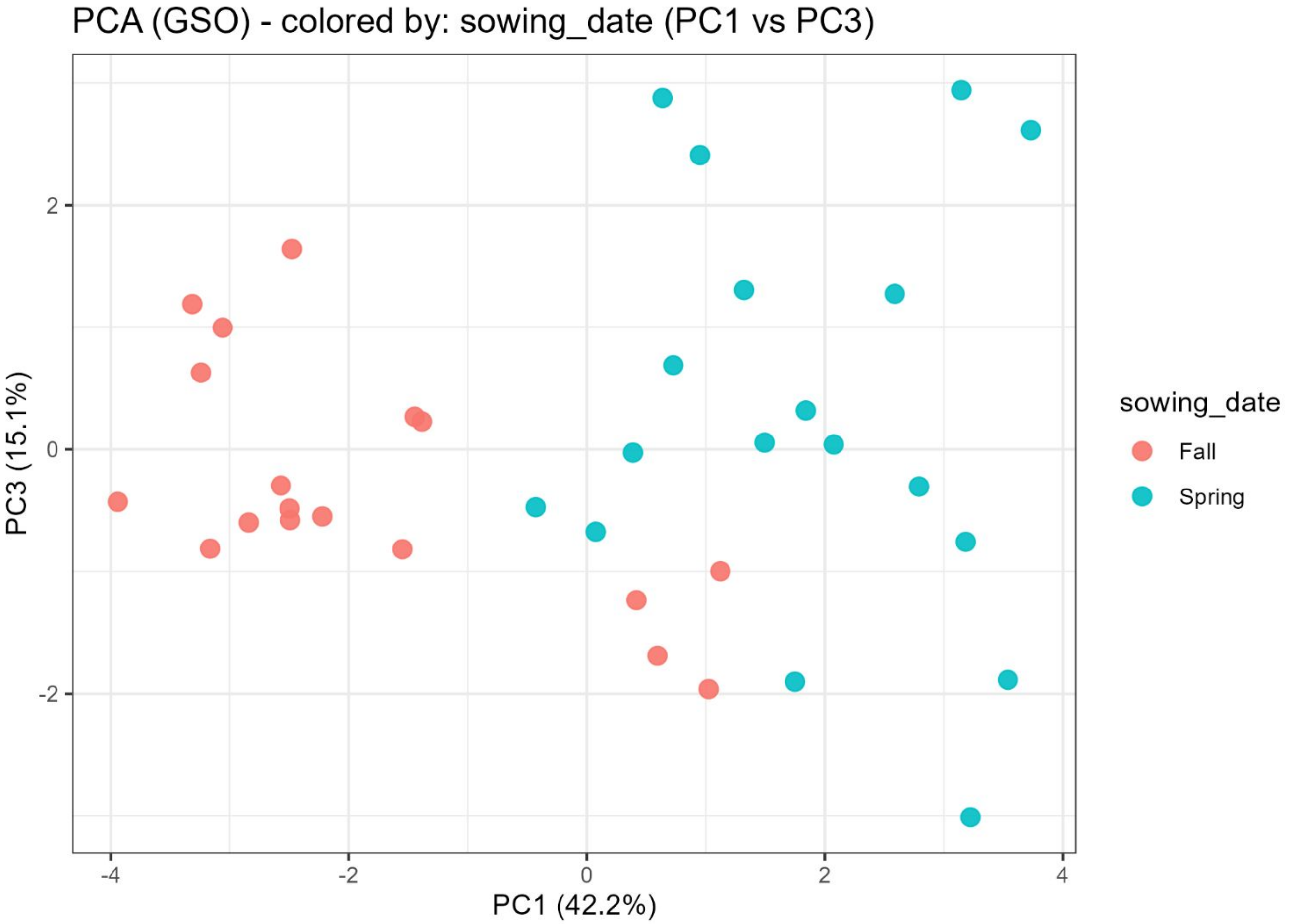

Figure S14. PCA score plot for the GSO trial (PC1 vs PC2) by fertilization. Scores of plot-level observations projected on PC1 (42.2%) and PC2 (19.6%) from PCA on standardized response variables. Points are colored by fertilization treatment (red, control; green, mineral; light blue, organic-mineral).

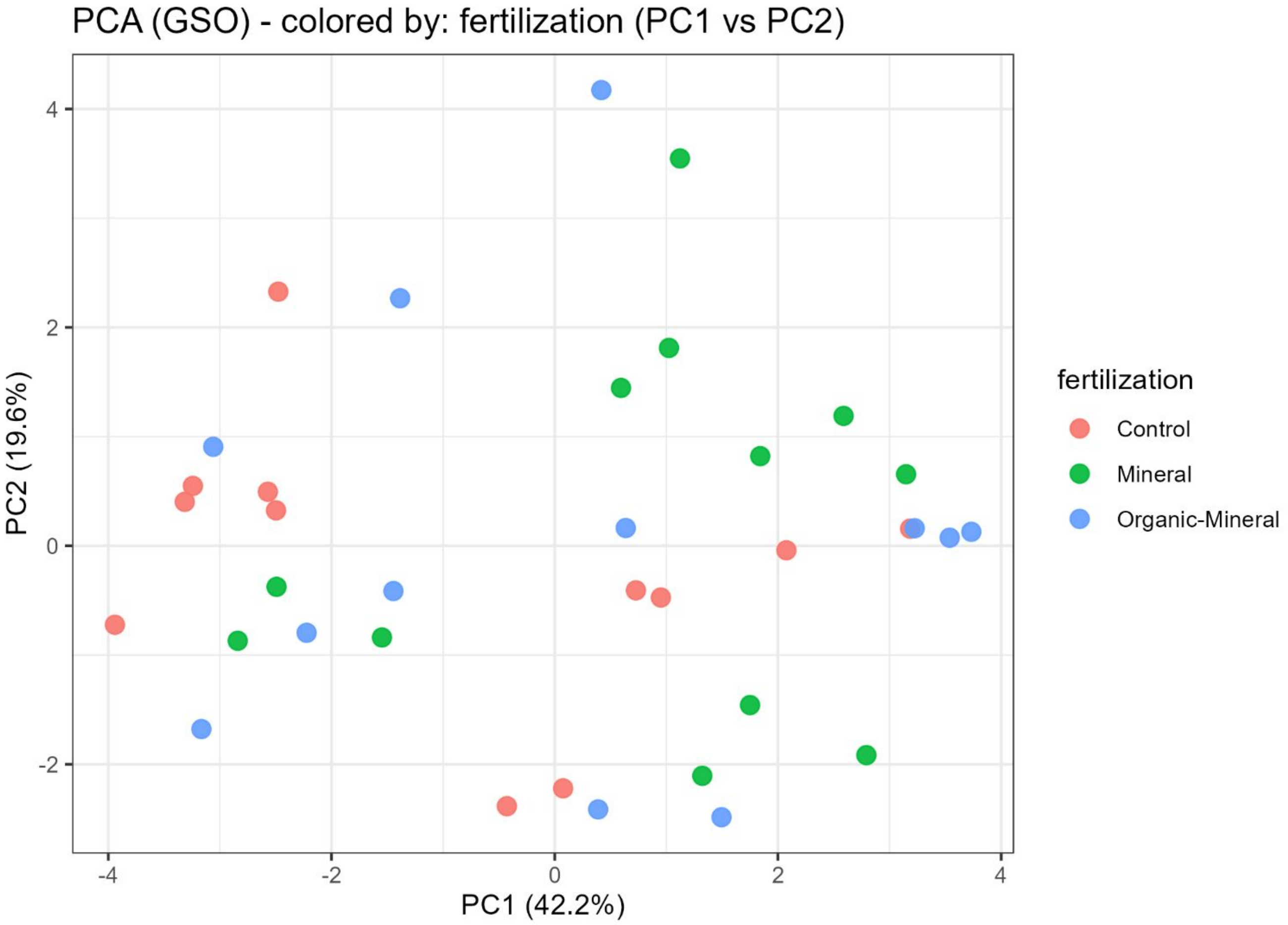

Figure S15. PCA score plot for the GSO trial (PC1 vs PC3) by fertilization. Scores of plot-level observations projected on PC1 (42.2%) and PC2 (15.1%) from PCA on standardized response variables. Points are colored by fertilization treatment (red, control; green, mineral; light blue, organic-mineral).

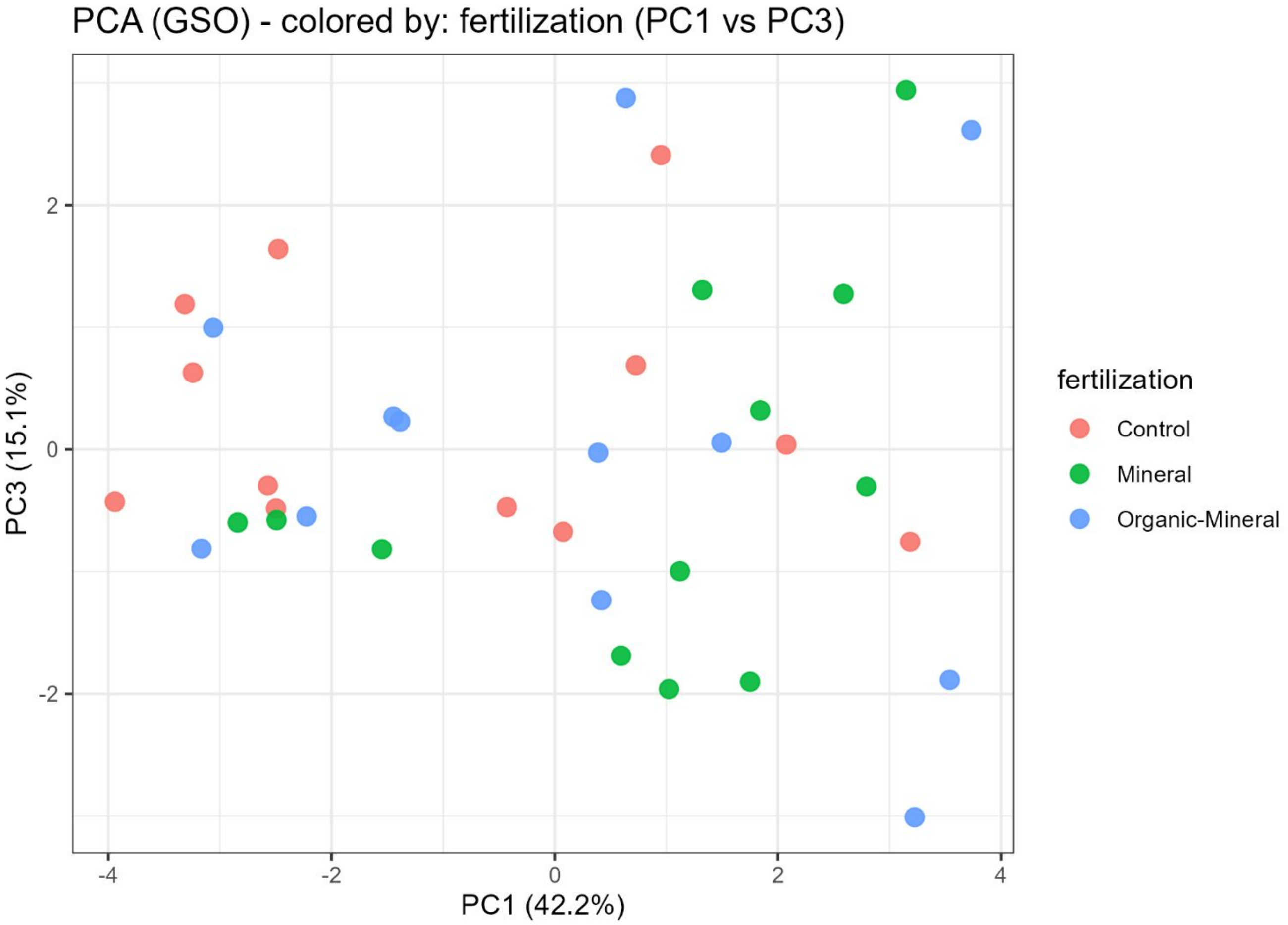

Figure S16. PCA score plot for the GSO trial (PC1 vs PC2) by genotype. Scores of plot-level observations projected on PC1 (42.2%) and PC2 (19.6%) from PCA on standardized response variables. Points are colored by genotype (red, Cannizzo; light blue, Saragolla).

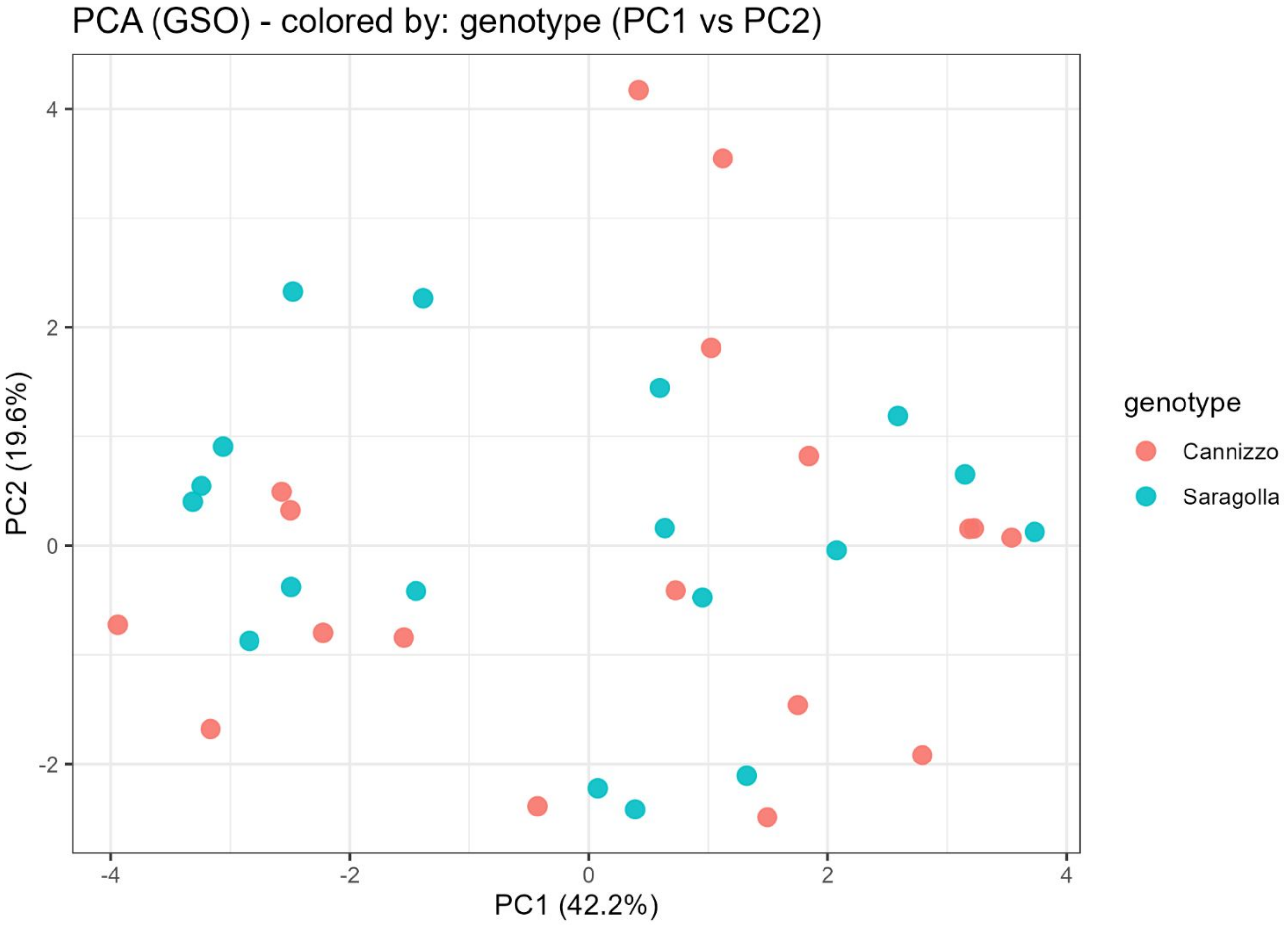

Figure S17. PCA score plot for the GSO trial (PC1 vs PC3) by genotype. Scores of plot-level observations projected on PC1 (42.2%) and PC2 (15.1%) from PCA on standardized response variables. Points are colored by genotype (red, Cannizzo; light blue, Saragolla).

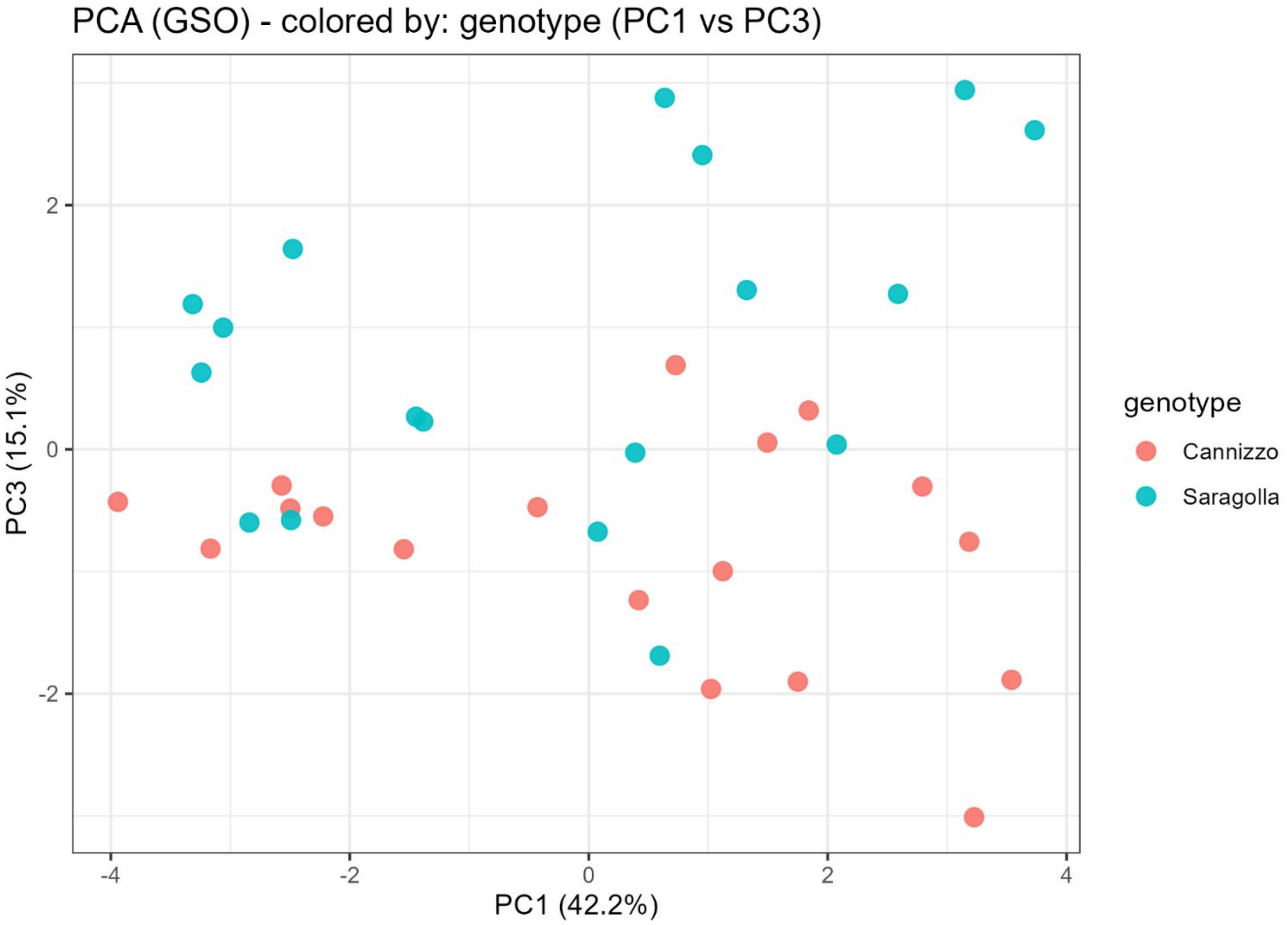

Supplement: Supplementary file 1 [file jf5c16192_si_001.pdf]
